# *Musa balbisiana* genome reveals subgenome evolution and functional divergence

**DOI:** 10.1038/s41477-019-0452-6

**Published:** 2019-07-15

**Authors:** Zhuo Wang, Hongxia Miao, Juhua Liu, Biyu Xu, Xiaoming Yao, Chunyan Xu, Shancen Zhao, Xiaodong Fang, Caihong Jia, Jingyi Wang, Jianbin Zhang, Jingyang Li, Yi Xu, Jiashui Wang, Weihong Ma, Zhangyan Wu, Lili Yu, Yulan Yang, Chun Liu, Yu Guo, Silong Sun, Franc-Christophe Baurens, Guillaume Martin, Frederic Salmon, Olivier Garsmeur, Nabila Yahiaoui, Catherine Hervouet, Mathieu Rouard, Nathalie Laboureau, Remy Habas, Sebastien Ricci, Ming Peng, Anping Guo, Jianghui Xie, Yin Li, Zehong Ding, Yan Yan, Weiwei Tie, Angélique D’Hont, Wei Hu, Zhiqiang Jin

**Affiliations:** 1 Key Laboratory of Biology and Genetic Resources of Tropical Crops, Institute of Tropical Bioscience and Biotechnology, Chinese Academy of Tropical Agricultural Sciences, Haikou, China; 2 Key Laboratory of Genetic Improvement of Bananas, Hainan province, Haikou Experimental Station, China Academy of Tropical Agricultural Sciences, Haikou, China; 3 BGI Genomics, BGI-Shenzhen, Shenzhen, China; 4 BGI Institute of Applied Agriculture, BGI-Shenzhen, Shenzhen, China; 5 CIRAD, UMR AGAP, Montpellier, France; 6 AGAP, Univ Montpellier, CIRAD, INRA, Montpellier SupAgro, Montpellier, France; 7 CIRAD, UMR AGAP, Guadeloupe, France; 8 Bioversity International, Montpellier, France; 9 CIRAD, UMR BGPI, Montpellier, France; 10 BGPI, CIRAD, INRA, Montpellier SupAgro, Montpellier, France; 11 Waksman Institute of Microbiology, Rutgers, The State University of New Jersey, Piscataway, NJ USA

**Keywords:** Next-generation sequencing, Phylogenetics, Genome duplication

## Abstract

Banana cultivars (*Musa* ssp.) are diploid, triploid and tetraploid hybrids derived from *Musa acuminata* and *Musa balbisiana*. We presented a high-quality draft genome assembly of *M. balbisiana* with 430 Mb (87%) assembled into 11 chromosomes. We identified that the recent divergence of *M. acuminata* (A-genome) and *M. balbisiana* (B-genome) occurred after lineage-specific whole-genome duplication, and that the B-genome may be more sensitive to the fractionation process compared to the A-genome. Homoeologous exchanges occurred frequently between A- and B-subgenomes in allopolyploids. Genomic variation within progenitors resulted in functional divergence of subgenomes. Global homoeologue expression dominance occurred between subgenomes of the allotriploid. Gene families related to ethylene biosynthesis and starch metabolism exhibited significant expansion at the pathway level and wide homoeologue expression dominance in the B-subgenome of the allotriploid. The independent origin of 1-aminocyclopropane-1-carboxylic acid oxidase (ACO) homoeologue gene pairs and tandem duplication-driven expansion of *ACO* genes in the B-subgenome contributed to rapid and major ethylene production post-harvest in allotriploid banana fruits. The findings of this study provide greater context for understanding fruit biology, and aid the development of tools for breeding optimal banana cultivars.

## Abstract (web summary)

A high-quality draft genome assembly of *Musa balbisiana* (the banana B-genome) is reported. Comparative genomic and gene functional analyses provide insights into the subgenome evolution and functional divergence between the A- and B-subgenomes.

## Main

Bananas (*Musa* ssp.) are large herbaceous plants that are perennial but monocarpic. They originated in Southeast Asia and the Western Pacific and were one of the first crops to be domesticated, about 7,000 years ago, in Southeast Asia^1,2^. Bananas are widely distributed throughout the tropics and subtropics, where they are a staple food and fruit for millions of people^2,3^. Moreover, bananas are one of the major export commodities of several developing countries and represent the largest international trade in fruit^3,4^. Thus, bananas are an essential food resource and have important socioeconomic and ecological roles.

The genus *Musa* belongs to the monocotyledon Musaceae family along with the genus *Ensete*. Its wild species have traditionally been subdivided into four sections: *Eumusa* (*x* = 11; *x* represents the number of chromosomes), *Rhodochlamys* (*x* = 11), *Australimusa* (*x* = 10) and *Callimusa* (*x* = 9 or 10)^5–7^, and refined recently to two sections in which the *Rhodochlamys* and *Australimusa* were merged into the *Eumusa* and *Callimusa*, respectively^8^. Most edible bananas belong to the *Eumusa* (or *Musa*) section, and are categorized into the dessert or cooking group based on their usage. Furthermore, bananas of this section are distinguished based on their genetic background as *Musa acuminata* (A-genome, 2*n* = 2*x* = 22; *n* represents the haploid chromosome number), *Musa balbisiana* (B-genome, 2*n* = 2*x* = 22), *Musa schizocarpa* (S-genome, 2*n* = 2*x* = 22) and *Australimusa* species (T-genome, 2*n* = 2*x* = 20)^2^. The majority of edible cultivated bananas originated from intraspecific or interspecific hybridization between wild diploid *M. acuminata* (A-genome) and *M. balbisiana* (B-genome) species. Combinations of these A- and B-genomes have resulted in various genotypes of cultivated edible bananas, including diploid (AA, BB and AB), triploid (AAA, AAB and ABB) and tetraploid (AAAB, AABB, ABBB) variants^6^. The triploid genotype variants constitute the predominant cultivated varieties that are planted worldwide.

Genome sequencing of the A-genome banana has provided insights into the evolution of monocotyledonous plants^1,9^. Although the A-genome represents a crucial step in the genetic improvement of banana, the lack of a high-quality B-genome sequence greatly hinders germplasm characterization and the molecular breeding of banana. A draft B-genome has previously been reported, but exhibited low quality, based on assembly and annotation via mapping reads to the A-genome^2^. Here, we sequenced the genome of the double haploid of the wild diploid genotype Pisang Klutuk Wulung (DH-PKW, 2*n* = 2*x* = 22), belonging to the species *M. balbisiana* that contributed the B-subgenome to cultivated allotriploid bananas. We further compared the B- and A-genomes to investigate subgenome evolution, genetic diversity and the functional divergence of subgenomes in polyploid bananas. Our analyses provide insights into the evolution and regulation of fruit-ripening processes in bananas. In particular, the results highlight a significant contribution of the B-genome towards ethylene biosynthesis and starch metabolism during fruit ripening.

## Results

### Genome assembly and annotation

**Fig. 1:**
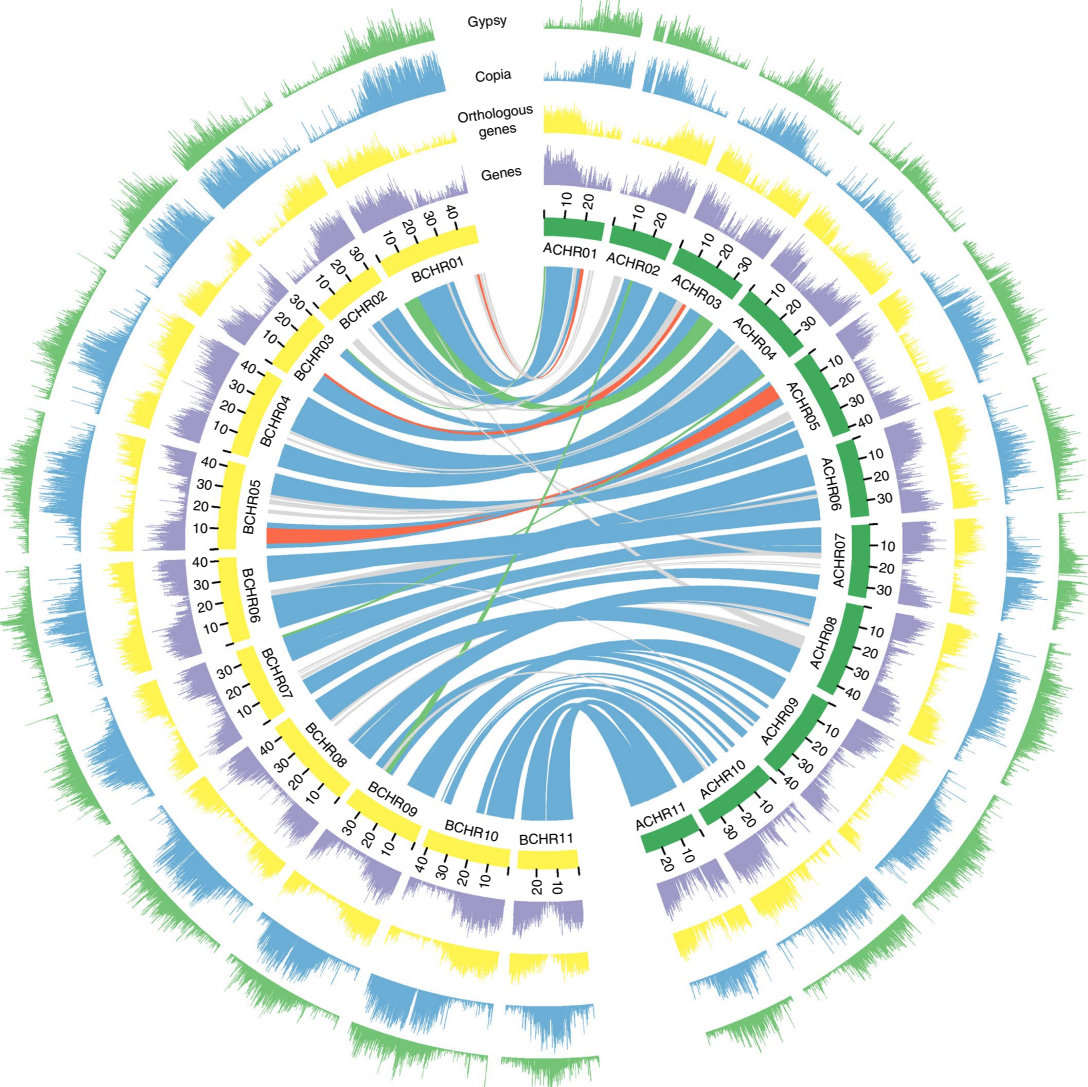
Characterization of *M. balbisiana* (B-genome) and *M. acuminata* (A-genome) chromosomes. Elements are arranged in the following scheme (from outer to inner). (1) Distribution of Gypsy elements (non-overlapping, window size, 50 kb); (2) distribution of Copia elements (non-overlapping, window size, 50 kb); (3) distribution of orthologous gene pairs between two genomes (non-overlapping, window size, 50 kb); (4) gene density (non-overlapping, window size, 50 kb); (5) syntenic relationships between A- and B-genomes. The connecting blue lines represent alignment blocks, red lines represent inversions, green lines represent translocations and grey lines show small blocks with<30 gene pairs.

To reduce heterozygosity, we used the DH-PKW genotype for our genome sequencing and assembly^10^. A total of 58.99 gigabases (Gb) (113×) of PacBio single-molecule long reads and 86.34 Gb (166×) Illumina paired-end and mate-pair reads were used for assembly (Supplementary Table 1), producing 492.77 megabases (Mb) of scaffolds. The contig N50 and scaffold N50 of the final assembly were 1.83 and 5.05 Mb, respectively (Supplementary Table 2). *K*-mer analysis suggested that the draft assembly covers approximately 95% of the genome size of DH-PKW (Supplementary Fig. 1). We further evaluated the completeness of the scaffold assembly using the BUSCO (v.3) plants datasets^11^. Precise exon placement of 91.3% of the total 1,440 single-copy orthologue groups in the embryophyta dataset was identified in the B-genome assembly. To anchor the scaffolds to chromosomes, we constructed high-throughput chromosome conformation capture (Hi-C) libraries of DH-PKW, generating 72 Gb (138×) Hi-C pair-end reads (Supplementary Table 1). Duplicate removal, sorting and quality assessment were performed with HiC-Pro^12^, and uniquely mapped valid reads were used for Hi-C scaffolding by LACHESIS software^13^ (Supplementary Fig. 2). As a result, 430 Mb (87.27%) of the assembly and 94.0% of the genes were placed on 11 chromosome groups (Fig. 1 and Supplementary Table 3). The 11 pseudo-molecules were named in accordance with the *M. acuminata* (A-genome) reference sequence^1,9^.

About 55.75% of the B-genome assembly was composed of repetitive sequences, which is higher than the 41.85% of the A-genome assembly (Supplementary Table 4). This may be due to the spanning of repetitive regions by long reads^14^. Long terminal-repeat (LTR) retrotransposons represented the most abundant fraction of transposable elements in the A- and B-genomes, among which the families Gypsy and Copia accounted for 12.88 and 28.04% of the B-genome, respectively. DNA transposons comprised 2.12% of the B-genome and 2.03% of the A-genome (Supplementary Table 4). LTR retrotransposons tended to accumulate near the centromeric and pericentromeric regions (Fig. 1). Active insertions of LTR retrotransposons occurred more recently in the B-genome (peak at 0–0.5 million years ago (MYA)) relative to the A-genome (peak at 1.5–2.0 MYA) after their divergence (Supplementary Fig. 3). Both genomes experienced a wave of LTR retrotransposon amplification but differed in insertion histories.

We annotated the genome using the MAKER pipeline^15^ incorporating ab initio predictions, homologous proteins and transcriptome data from six samples, resulting in 35,148 protein-coding genes in the B-genome (Supplementary Table 5). Of these, 33,137 (94.27%) were located on the 11 pseudo-chromosomes (Supplementary Table 3). Overall, 86% of the genes transcribed in our RNA sequence (RNA-Seq) analysis. Additionally, we identified 3,329 transcription factors among 88 families in the B-genome using the iTAK programme^16^ (Supplementary Table 6).

### The B-genome is more sensitive to fractionation than the A-genome

Compared to other monocots, the *Musa* lineage exhibits a relatively slower evolutionary rate as demonstrated previously^17^ (Supplementary Fig. 4). Phylogenetic analyses based on 519 single-copy orthologous genes indicated a very recent divergence time of about 5.4 MYA for the A- and B-genomes, with their common ancestor having diverged from Poaceae ~134 MYA (Supplementary Fig. 5). This estimate is consistent with a divergence time of 4.6 MYA between the A- and B-genomes, which was estimated based on 17 bacterial artificial chromosome (BAC) clones that contained 23.5 Kb of coding sequence^18^. Our estimate is more recent than the 20.9 MYA (estimated by three genes and one internal transcribed spacer region) or the 27.9 MYA (estimated by 19F genes)^19,20^. However, the increased sampling of informative characters in our genome-wide study for phylogenetic analyses should contribute to a more accurate divergence time estimation.

Three rounds of whole-genome duplication (WGD) events (α-, β- and γ-WGD) have occurred in the *Musa* lineage^1^, which was validated by our four-fold synonymous third-codon transversion (4dTv) analysis (Supplementary Fig. 6). WGD is frequently, and almost always, followed by diploidization and fractionation, which includes chromosome rearrangement, gene loss and biased retention^21^. After diploidization and divergence, A- and B-genomes start to evolve independently. To investigate the evolutionary differences between the A- and B-genomes after divergence, we performed three types of comparative analysis: (1) assessment of gene family expansion/contraction between A- and B-genomes by comparison to 14 other plant genomes; (2) comparison of structural variation in the A- and B-genomes by synteny analysis; and (3) comparison of synteny between the ancestral syntenic block and the A/B-genomes.

We analysed the gene family clustering and expansion/contraction of banana genomes of *M. acuminata* (A-genome) and *M. balbisiana* (B-genome), compared to 14 other plant genomes using OrthoMCL and CAFE^22,23^ (Supplementary Table 7 and Supplementary Fig. 7). We found 9,038 gene families that were conserved in *M. balbisiana*, *M. acuminata*, *Oryza sativa*, *Brachypodium distachyon* and *Vitis vinifera*. In contrast, 348 and 639 gene families were specific to the A- and B-genome, respectively (Supplementary Fig. 8). After their divergence, 1,761 gene families were expanded and 203 were contracted in the A-genome, while 392 gene families were expanded and 1,008 contracted in the B-genome (Supplementary Fig. 9 and Supplementary Tables 8 and 9). Kyoto Encyclopedia of Genes and Genomes (KEGG) pathway enrichment analysis of the significantly expanded gene families (*P* < 0.05) in the B-genome suggested that it was enriched in the photosynthesis and biosynthesis of secondary metabolite pathways, including those associated with the metabolism of inositol, starch and sucrose, linoleic acid and arachidonic acid (Supplementary Fig. 10 and Supplementary Table 10). Plants produce a high diversity of secondary metabolites with prominent functions in defence against a variety of herbivores and pathogens, in addition to the mitigation of various types of abiotic stresses^24^. Thus, these observations are consistent with the association of the B-genome with improved vigour and tolerance to both biotic and abiotic stresses^2^.

Synteny analysis indicated a high level of genomic colinearity and sequence similarity between the A- and B-genomes (Supplementary Fig. 11). We identified 72 large syntenic blocks between the A- and B-genomes, including 15 large blocks each containing over 900 gene pairs. These 72 syntenic blocks comprised 75.02% of A-genome space (containing 23% transposable elements) and 68.01% of B-genome space (containing 22% transposable elements) (Supplementary Table 11). We also identified two large translocations and two inversions between the A- and B-genome after their divergence. One large reciprocal translocation comprises 7.09 Mb on chromosome (chr)1 of the B-genome and 7.03 Mb on chr:3 of the A-genome, and one large inversion of 9.39 Mb on chr 5 of the B-genome and 8.83 Mb on chr 5 of the A-genome (Fig. 1 and Supplementary Fig. 11). These translocations and inversions were also supported by the rearrangements based on genetic mapping^25^, and can serve to introduce novel genetic diversity into the A- and B-genomes^26^.

Previously, 12 *Musa* ancestral blocks were assembled that represented the ancestral genome before α/β-WGD^1,27,28^. We identified 97 syntenic blocks resulting from α/β-WGD events in the B-genome by comparison to the 12 *Musa* ancestral blocks. These blocks contained 24,639 genes and represented 70.10% of all gene models involved in WGD-driven regions. We also identified 100 syntenic blocks that contained 26,780 genes (75.61%) in the A-genome (Supplementary Table 12). Of these ancestral *Musa* α/β blocks, 56.89% (15,236) and 60.59% (14,930) were singletons in the A- and B-genome, respectively, suggesting that genome fractionation (gene loss) and diploidization processes had occurred extensively after WGD events in the *Musa* lineage (Supplementary Table 12).

Taken together, our results indicated that the B-genome exhibited less expansion and more contraction of gene families, less syntenic coverage ratio and a higher singleton ratio in the ancestral blocks compared to the A-genome after divergence. Cycles of WGD followed by diploidization and fractionation have occurred across land plants, and are important in determining chromosome structure and gene content. Consequently, these processes have significantly contributed to the evolutionary success of plants^21,29^. The diploidization and fractionation processes involve a series of evolutionary events, including repetitive DNA loss, chromosome rearrangements and complex patterns of gene loss^29,30^. The above evidence supports the hypothesis that the B-genome was more sensitive to fractionation than the A-genome after their divergence.

### Genetic diversity in polyploid bananas and functional divergence of subgenomes

Polyploid species usually exhibit vigorous growth, including high-quality production and high fitness^31^. Most banana cultivars are polyploid and exhibit various levels of ploidy and genomic background^32^. The genetic classification of some bananas is discordant, as is the case for Pelipita. Previous studies have shown that its karyotype comprises eight A and 25 B chromosomes as opposed to the predicted 11 A and 22 B chromosome distribution^33^. Understanding the genetic diversity and genomic constitution of *Musa* accessions would inform genomic group classifications, in addition to conservation and breeding strategies. Therefore, we re-sequenced five triploid bananas and four diploid bananas to investigate their genetic diversity (Supplementary Table 13).

**Fig. 2:**
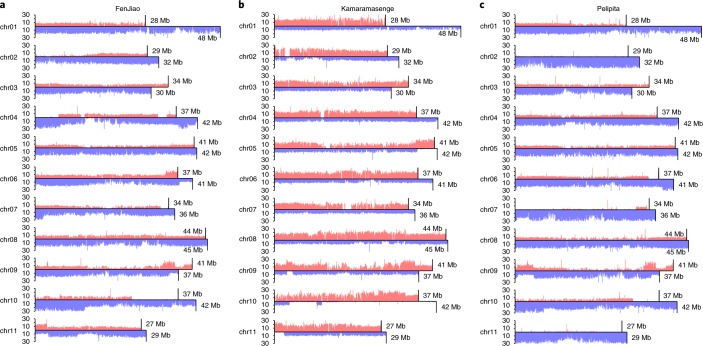
Coverage depth and genome structure summary for three allotriploid banana accessions. **a**–**c**, Chromosome coverage and structure for accessions FenJiao (genome group, ABB) (**a**), Kamaramasenge (genome group, AAB) (**b**) and Pelipita (genome group, ABB) (**c**) with 100 kb non-overlapping sliding windows. The upper red bar and lower blue bar represent coverage depth of the A- and B-subgenome, respectively.

Simultaneous alignment of re-sequencing data to the A- and B-subgenomes identified the uniquely mapped reads that were used to analyse coverage depth, variations calling and homoeologous exchanges on each chromosome (Supplementary Table 14). Homoeologous exchanges were characterized by read coverage that showed a chromosomal region with a duplicated copy from the corresponding homoeologous subgenome^34^. These analyses confirmed that genome constitutions for the banana accessions were, in most cases, consistent with previous genome group classifications based on morphological traits. We identified 48 segmental homoeologous exchanges in the accession FenJiao, including nine from the B- to the A-subgenome and 39 in the reverse direction (Fig. 2a and Supplementary Table 15). We also found four segmental homoeologous exchanges from the B- to the A-subgenome in the accession Kamaramasenge, and replacement of chromosome 10 of the B-subgenome by the A-subgenome. (Fig. 2b and Supplementary Table 15). For the accession Pelipita, chromosomes 2, 7 and 11 of the A-subgenome were replaced by the B-subgenome and there were 18 segmental homoeologous exchanges on chromosomes 6, 9 and 10 (Fig. 2c and Supplementary Table 15). This indicates the eight A and 25 B chromosome constitution of Pelipita, consistent with previous genomic in situ hybridization studies^33^. This classification is further supported by phylogenetic analyses based on genotyping data (Supplementary Fig. 12). A total of 18,475,661 single-nucleotide polymorphisms (SNPs), 1,425,391 small insertions and deletions and 220,452 structural variations were identified in the samples (Supplementary Tables 16–18). Analysis of gene and SNP density on the chromosomes indicated that SNPs were preferentially located on non-gene-rich regions (Supplementary Fig. 13). There were ~2.5-fold SNPs on the A-genome of Pisang_Mas and Pisang_Kra compared to the B-genome of Balbisiana (Supplementary Table 16). The nucleotide diversity (π,0.0059) of A-subgenomes was higher than that of the B-subgenomes (0.0031) in accessions Fenjiao, Pelipita and Kamaramasenge.

Gene family expansion and contraction analysis of *M. acuminata* and *M. balbisiana* in comparison to other sequenced genomes indicated that there are 83 gene families significantly expanded in the A-genome (and conversely contracted in the B-genome). These families included plant–pathogen interactions, glycosphingolipid biosynthesis-ganglio series and glycosaminoglycan degradation pathways among others (Supplementary Table 19). Conversely, 33 gene families were significantly expanded in the B-genome (and contracted in the A-genome). These families included those involved in photosynthesis, metabolic pathways and ribosome, among others (Supplementary Table 20). This indicates that the A- and B-genomes may have functionally diverged at the genome level during their respective genome evolution.

To explore the transcription of allopolyploid subgenomes, we assessed the expression of homoeologue genes from the A- and B-subgenomes of the triploid FenJiao. Expression levels were measured within different tissues, at different stages of fruit development and ripening and in banana seedlings after abiotic stress treatments (Supplementary Table 21). A total of 25,717 homoeologous gene pairs were identified between the A- and B-subgenomes based on the gene alignment method with cumulative identity percentage (CIP) ≥ 60% and cumulative alignment length percentage (CALP) ≥ 60% (refs. ^35,36^). Among the homoeologue gene pairs, 81.83% were further supported by syntenic analysis (Supplementary Table 22). Expression of all homoeologue gene pairs was assessed to determine the distribution of expression fold change of B/A in the triploid ‘FJ’. The log_2_(RPKM B/RPKM A) was 1.2/1, where RPKM is reads per kilobase million, which differed from the genomic constitution value of 2/1 (Supplementary Fig. 14). This result could be explained by dosage compensation, wherein the triploid expression levels are reduced to a diploid state^37,38^. We further characterized 1,075 and 4,032 homoeologue gene pairs that showed expression dominance in the A- and B-subgenome, respectively (Supplementary Table 23). KEGG enrichment analysis indicated that genes with expression dominance in the B-subgenome were associated with 2-oxocarboxylic acid metabolism and the arginine biosynthesis pathway (*q*-value < 0.05) (Supplementary Table 24), whereas those showing expression dominance in the A-subgenome were not significantly enriched in KEGG pathways.

Non-synonymous/synonymous substitution (Ka/Ks) ratios were calculated for all homoeologue gene pairs between the A- and B-subgenomes. The Ka/Ks ratios of genes with expression dominance in the A-subgenome (median, 0.157) were slightly lower than those in the B-subgenome (median, 0.196) and non-dominant genes (median, 0.186) (Supplementary Fig. 15).

We then constructed a gene co-expression network for those genes with expression dominance using weighted gene co-expression network analysis (WGCNA)^39^. The results indicated that 87 and 295 genes with dominance expression interacted with 4,302 and 4,612 genes in the A- and B-subgenome, respectively. KEGG pathway enrichment analysis suggested that genes in the co-expression network of the A- and B-subgenomes were commonly associated with starch and sucrose metabolism (ko00500) and other metabolic pathways. In particular, ubiquinone and other terpenoid-quinone biosynthesis, photosynthesis–antenna proteins, carotenoid biosynthesis and other glycan degradation pathways were specially enriched in the A-subgenome (*q*-value < 0.05) (Supplementary Fig. 16 and Supplementary Table 25). In contrast, selenocompound metabolism and cyanoamino acid metabolism pathways were particularly enriched in the B-subgenome (*q*-value < 0.05) (Supplementary Fig. 17 and Supplementary Table 26). Overall, these results further support the hypothesis of functional divergence between the A- and B-genomes at the transcriptional level.

### Expression dominance of homoeologue gene pairs in the ethylene biosynthesis pathway and expansion of the ACO family affect fruit ripening

**Fig. 3:**
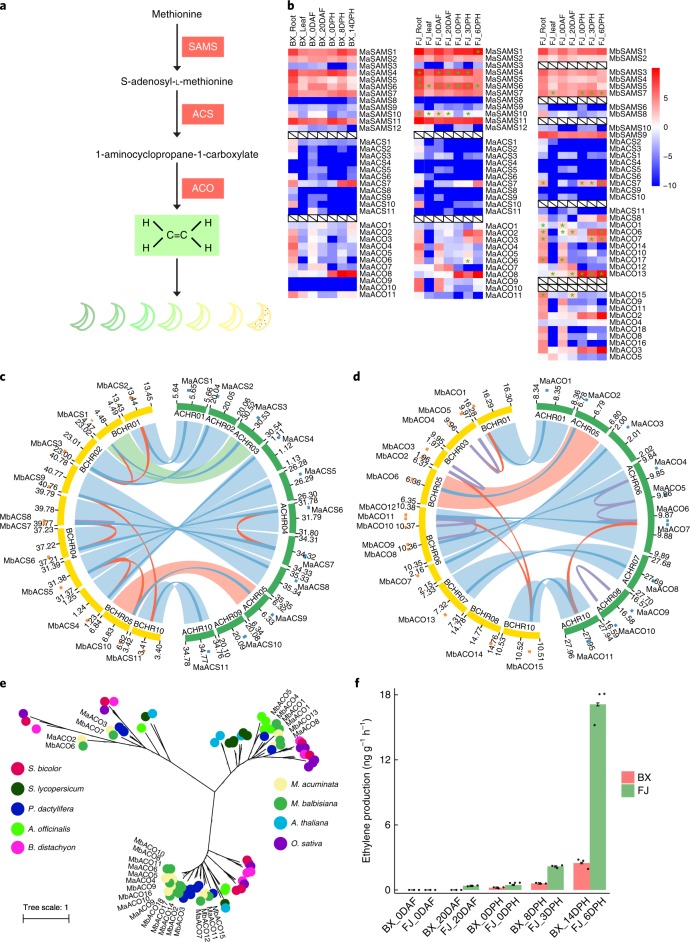
Phylogeny and expression patterns of ethylene biosynthesis genes between *M. acuminata* (A-genome) and *M. balbisiana* (B-genome). **a**, Overview of the ethylene biosynthesis pathway. **b**, Expression patterns of SAMS, ACS and ACO family genes in the root and leaf, and at different stages of fruit development and ripening in BX, the A-subgenome of FJ and the B-subgenome of FJ. Genes aligned horizontally in the heat map indicate homoeologue gene pairs between the A- and B-genomes. White boxes with diagonals indicate the lack of homoeologue gene pairs between the A- and B-subgenomes. Asterisks indicate expression dominance of homoeologue gene pairs between the A- and B-subgenomes of FJ. **c**,**d**, Synteny analysis of ACS (**c**) and ACO (**d**) families between the A- and B-genomes. Red lines indicate paralogous gene pairs resulting from WGD, blue lines indicate homoeologous gene pairs, purple lines indicate tandem duplication, light blue strips indicate aligned syntenic blocks, light green strip indicates translocation block and light red strips indicate inversion blocks.The blocks in outer ring represent location and length of genes; blue blocks represent genes from A-genome and orange blocks represent genes from B-genome. **e**, Phylogenetic analysis of ACO family genes among nine species: *M. acuminata*, *M. balbisiana*, *A. thaliana*, *O. sativa*, *Sorghum bicolor*, *Solanum lycopersicum*, *Phoenix dactylifera*, *Asparagus officinalis* and *B. distachyon*. **f**, Ethylene production at different stages of fruit development and ripening in BX and FJ. Error bars show standard error of the mean from three independent experiments (*n* = 3).

Ethylene plays a key role in the regulation of climacteric fruit ripening post-harvest^40^. The core enzymatic steps in the ethylene biosynthesis pathways are well characterized, and include S-adenosyl-l-methionine synthase (SAMS), 1-aminocyclopropane-1-carboxylic acid synthase (ACS) and ACO^41,42^ (Fig. 3a). However, the expansion and expression dominance of these homoeologue gene pairs during polyploid fruit ripening remains largely unknown.

We identified 12 SAMS, 11 ACS and 11 ACO genes from the A-genome and 10 SAMS, 11 ACS and 18 ACO genes in the B-genome, which represents a significant expansion compared to the seven other sequenced plant species among the monocots and eudicots^22^ (Supplementary Tables 27 and 28). We further characterized 28 homoeologue gene pairs from the A- and B-genomes (Supplementary Table 29). These gene pairs displayed similar expression profiles in the BaXiJiao (*Musa* AAA group, cv. Cavendish, BX), the A-subgenome of FenJiao (*Musa* ABB group, cv Pisang Awak, FJ) and the B-subgenome of FJ (Fig. 3b and Supplementary Tables 30–32). Interestingly, eight gene pairs exhibited homoeologue expression dominance in the B-subgenome and five gene pairs were dominantly expressed in the A-subgenome of FJ (Fig. 3b and Supplementary Tables 33 and 34). The dominant expression of these genes in various tissues may be related to the fundamental role of ethylene biosynthesis.

Both ACS and ACO have previously been demonstrated as limiting enzymes within ethylene biosynthesis^41^. The expression abundance of *MA-ACS1* (AB021906) and *MA-ACO1* (X91076) is consistent with ethylene production during the post-harvest banana-ripening period^43^. Of the ten *ACS* gene pairs, *MaACS7*/*MbACS7*, which is a homologue of *MA-ACS1*, exhibited high expression levels during fruit ripening and was dominantly expressed in the B-genome (Fig. 3b). *MbACS6* and *MbACS7* are paralogous in a large syntenic block (the block contains 19 gene pairs), and maintain synteny and close evolutionary relationships to *MaACS6* and *MaACS7*, respectively, suggesting that these genes duplicated from WGD (Fig. 3c and Supplementary Fig. 18). Of the nine *ACO* gene pairs, three (*MaACO2*/*MbACO6*, *MaACO3*/*MbACO7* and *MaACO8/MbACO13*) exhibited high expression levels during fruit ripening and were dominantly expressed in the B-subgenome (Fig. 3b). *MaACO2*/*MbACO6* and *MaACO3*/*MbACO7* are in the syntenic block of chr:5 and chr:6 between the A- and B-genome, respectively, and belong to the same phylogenetic clade (Fig. 3d,e and Supplementary Table 35). These results indicated that *MaACO2*/*MbACO6* and *MaACO3*/*MbACO7* originated independently and developed crucial functions during fruit ripening.

We also observed high expression levels (log_2_RPKM > 4 in at least one stage of fruit ripening in the B-genome) of homoeologue gene pairs, probably related to fruit softening (pectin methylesterases, galactosidases, expansions and pectatelyase)^44^, cell wall modification (xyloglucan endotransglucosylase/hydrolases, fasciclin-like arabinogalactan proteins and β-d-xylosidase)^45,46^ and aroma production (alcohol dehydrogenases)^40^. These genes are closely involved in fruit ripening and are regulated by ethylene^44^. Almost all of these gene pairs showed expression dominance in the B-subgenome of FJ during fruit ripening, which is the same as the dominance expression of gene pairs related to ethylene biosynthesis (Supplementary Fig. 19 and Supplementary Tables 36 and 37). This co-dominance of homoeologue gene pairs in the B-subgenome further supports the significant contribution of the B-genome to ethylene biosynthesis and fruit ripening.

Gene duplication is a major mechanism that generates new genetic diversity as a basis for evolutionary innovation in eukaryotes^47^. Compared to the 11 *ACO* genes within the A-genome, the *ACO* genes in the B-genome expanded significantly to 18 members. The expansion of *ACO* genes, including *MbACO2*, *-3*, *-4*, *-5* in chr 3, *MbACO8*, *-9*, *-11* in chr 6 and *MbACO16*, *-18* in scaffolds, was driven by tandem duplications in the B-genome (Fig. 3b,d). Of the *ACO* genes that were expanded in the B-genome, *MbACO2* and *MbACO3* showed strong expression levels during the fruit-ripening stages with log_2_RPKM > 11 at 6 days post-harvest (DPH) in FJ, which is coincident with the ethylene climacteric period (Fig. 3b,f and Supplementary Table 38). In addition, *MbACO8*, *-9*, *-11*, *-16*, *-18* exhibited high expression levels in roots and fruits at 0 days after flowering (DAF) (Fig. 3b and Supplementary Table 38). These genes belonged to the same cluster and their expression patterns were highly concordant with their duplication (Fig. 3d,e), suggesting that the expansion and evolution of *ACO* genes in the B-genome contributed to tissue development and fruit ripening.

Ripening of FJ was more rapid than BX during post-harvest ripening. BX required 8 and 14 DPH to reach the more-green-than-yellow and full-yellow stages, respectively, whereas FJ required 3 and 6 DPH to reach these stages, respectively (Supplementary Fig. 20). The dominant expression of ethylene biosynthesis and fruit ripening-related gene pairs and the expansion of the ACO family in the B-genome could have contributed to increased ethylene production and faster fruit ripening in FJ compared to BX.

### The active starch metabolic pathway in the B-genome during fruit development and the ripening process

**Fig. 4:**
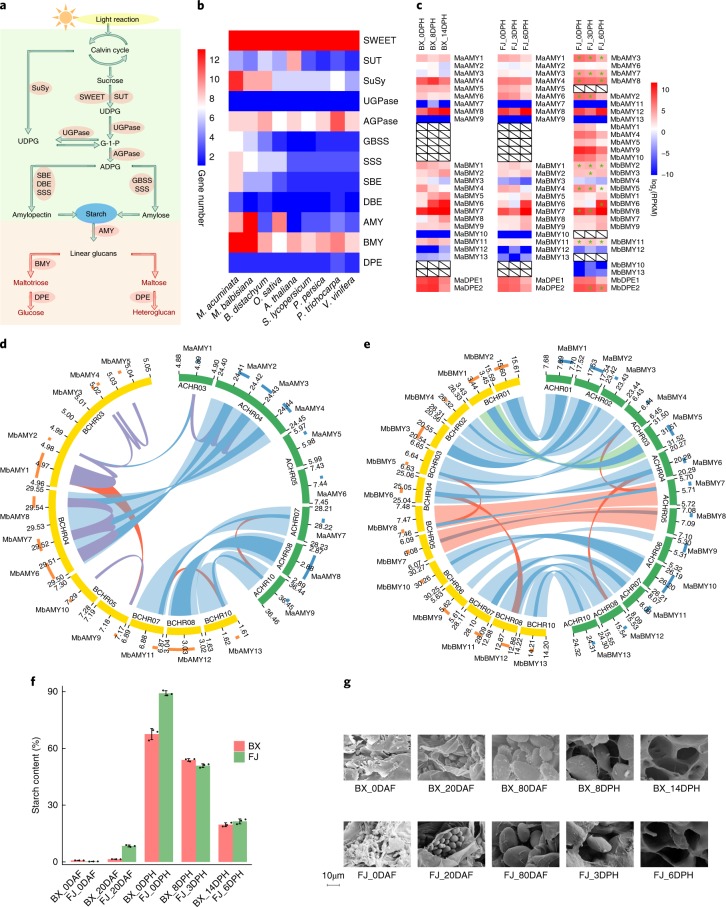
Comparison of genomic expansion, evolutionary history and differential expression patterns of the starch metabolic pathway between *M. acuminata* (A-genome) and *M. balbisiana* (B-genome). **a**, Overview of the starch biosynthesis and degradation pathway. **b**, Gene families in the starch metabolic pathway that are expanded in *M. acuminata* and *M. balbisiana*. **c**, Expression patterns of families AMY, BMY and DPE in the starch degradation pathway in BX, the A-subgenome of FJ and the B-subgenome of FJ during fruit-ripening stages. Horizontally oriented genes in the heat map indicate homoeologue gene pairs between the A- and B-genomes. White boxes with diagonals indicate that no homoeologue gene pairs were identified between the A- and B-genomes. Asterisks indicate expression dominance of homoeologue gene pairs between the A-subgenome of FJ and the B-subgenome of FJ. **d**,**e**, Synteny analyses of AMYs (**d**) and BMYs (**e**) between the A- and B-genomes. Red lines indicate paralogous gene pairs resulting from segmental/WGD-driven duplication, blue lines indicate homoeologous gene pairs, purple lines indicate tandem duplication, light blue strips indicate aligned syntenic blocks, light green strip indicates translocation block and light red strips indicate inversion blocks. The blocks in the outer ring represent location and length of genes; blue blocks represent genes from A-genome and orange blocks represent genes from B-genome. **f**, Starch contents at different stages of fruit development and ripening in BX and FJ. Error bars show standard error of the mean from three independent experiments (*n* = 3). **g**, Scanning electron microscopy of starch granules at different stages of fruit development and ripening in BX and FJ. The experiment was repeated three times independently with similar results.

Starch is the most widespread and abundant storage carbohydrate in plants. It is also a major component of cultivated banana, accumulating to high levels (~60–75% of dry weight) and leading to the presence of large starch granules (~8–30 m) during banana fruit development, along with near-complete conversion to soluble sugars during post-harvest ripening^48–51^. Thus, banana could serve as an excellent model for the investigatation of starch metabolism in fresh starchy fruits. The major enzymes that are responsible for starch biosynthesis (sugars will eventually be exported transporter: SWEET; sucrose transporter: SUT; sucrose synthase: SuSy; UDP-glucose pyrophosphorylase: UGPase; ADP-glucose pyrophosphorylase: AGPase; granule-bound starch synthase: GBSS; soluble starch synthase: SSS; starch branching enzyme: SBE; and starch debranching enzyme: DBE) and degradation (α-amylase: AMY; β-amylase: BMY; and starch phosphorylase: DPE) are encoded by multigenic families^51–56^ (Fig. 4a). We identified 101 starch metabolism-related genes in the A-genome, including 77 in the starch synthesis pathway and 24 in the starch degradation pathway. Ninety-six such genes were identified in the B-genome, including 68 in the starch synthesis pathway and 28 in the starch degradation pathway (Supplementary Table 39).

In the starch synthesis pathway, five (SuSy, GBSS, SSS, SBE and DBE) out of nine families showed obvious expansion in the A- and B-genomes of banana, compared to seven other plant species that included two fruit plants, tomato and grape. These expansions suggest a potential significance in banana (Fig. 4b and Supplementary Table 40). We characterized 54 homoeologue gene pairs from these families in the A- and B-genomes. Of these, 27 homoeologue gene pairs had expression dominance in root, leaf and fruit tissues with seven dominant in the A-subgenome and 20 in the B-subgenome (Supplementary Fig. 21 and Supplementary Tables 41–45). Consequently, the starch synthesis pathway is more active in different tissues within the B-subgenome than in the A-subgenome. Of those gene pairs with dominant expression in the B-subgenome, *MbSWEET17*, *MbSuSy1*, *MbSuSy2* and *MbSuSy9* had high expression levels (log_2_-based RPKM > 6) at 0 DAF and 20 DAF, suggesting an important role in starch synthesis during fruit development (Supplementary Fig. 21 and Supplementary Table 43). In contrast, most of the starch synthesis-related genes that were unique to the A- or B-genome exhibited low expression levels (Supplementary Fig. 22).

Within the starch degradation pathway, genome annotation indicated that families AMY and BMY had an obvious expansion in the banana A- and B-genomes compared to seven other plant species (Fig. 4b and Supplementary Table 40). We characterized 21 homoeologue gene pairs within this pathway from the A- and B-genomes. Among these gene pairs, 11 had dominant expression and were associated with the B-subgenome during fruit ripening (Fig. 4c and Supplementary Tables 46–50). Among the dominant genes, *MbAMY-2, MbAMY-3*, *MbAMY-8*, *MbBMY-6*, *MbBMY-8* and *MbDPE-2* exhibited high expression (log_2_-based RPKM > 6 in at least one stage) during fruit ripening (Fig. 4c and Supplementary Table 48). *MbAMY-1*, *-2*, *-3*, *-4*, *-5* and *MbAMY-6*, *-7*, *-8* showed close proximity and an evolutionary relationship, suggesting that these genes duplicated from a tandem copy (Fig. 4d and Supplementary Fig. 23). *MbBMY-5*, *-8* and *MbBMY-6*, *-12* are two paralogous pairs in syntenic blocks of the B-genome and showed close evolutionary relationships, suggesting that these genes duplicated from WGD (Fig. 4e and Supplementary Fig. 24). Taken together, these results suggest that tandem-driven *AMY* duplication and WGD-driven *BMY* duplication in the B-genome contribute to starch degradation.

The dominant expression of genes involved in starch biosynthesis in the B-subgenome could have led to increased starch accumulation during fruit development in FJ relative to BX (Fig. 4f,g and Supplementary Fig. 21). In addition, the dominant expression of genes related to starch degradation in the B-subgenome also probably led to elevated starch degradation in FJ (Fig. 4c,f,g). Therefore, the active starch metabolic pathway in the B-genome leads to marked starch accumulation and degradation during the fruit development and ripening processes, respectively.

## Discussion

Most cultivated bananas are triploid, having evolved from two wild diploid species, *M. acuminata* and *M. balbisiana*^6^. Our previous *M. acuminata* genome sequencing efforts provided genomic resources to inform banana breeding^1^. However, there is also an urgent need to develop the genomic resources of *M. balbisiana*, which is a crucial contributor to cultivated varieties. Improved understanding of the B-genome structure, subgenome evolution, homoeologous exchange, genetic diversity in polyploidy bananas and gene expansion and expression patterns will help in the design and application of breeding strategies for novel banana cultivars with improved traits.

Cycles of WGD followed by diploidization events have occurred across land plants, and have significantly contributed to their evolutionary success^24,25^. Our results indicate that the B-genome may be more sensitive to fractionation than the A-genome after WGD, although the A- and B-subgenomes have diverged very recently. Variation in genomic structure between the A- and B-genomes consists of chromosome rearrangements and gene loss during diploidization, which has resulted in the functional divergence of subgenomes in polyploidy bananas. This divergence is supported by differential enrichment of expanded/contracted gene families between the A- and B-genomes and the expression dominance of homoeologue genes from A- and B-subgenomes in triploids. Although homoeologue expression dominance has been identified in certain polyploid species^57–60^, the relationship between homoeologue expression dominance and functional divergence (especially in regard to ethylene biosynthesis/starch metabolism) of subgenomes in triploids remains to be elucidated. Thus, these results provide an important basis for the improvement of agriculturally important traits by focusing selection on transcriptionally dominant genes. It is worth noting that homoeologous exchanges may obscure the signal of expression dominance in subgenomes of allopolyploids^61^. The extensive homoeologue exchanges in allopolyploid bananas may remove many progenitor genome conflicts that result in subgenome biases in gene content and expression. Thus, homoeologous exchanges may contribute to the diversity of homoeologue expression dominance and induce a series of rapid genetic and epigenetic modifications for agronomic traits^61^.

Previous studies have suggested an important role of ethylene production in fruit ripening and starch metabolism with regard to fruit quality post-harvest^27,43,55^. However, the genetic mechanisms underlying polyploid fruit ripening are less well known. Here, we analysed biological processes related to these two pathways in triploid bananas. We identified significant genomic expansions and dominant expression of homoeologue genes in the B-genome at the pathway level of gene families and, most notably, in the ACO family, known to play a critical role in ethylene production. Our analysis revealed the origin and evolution of crucial gene families in these pathways, particularly for the independent origin of the *MaACO2*/*MbACO6* and *MaACO3*/*MbACO7* gene pairs and their specific function in fruit ripening. Moreover, we identified that this tandem event has led to expansion of *ACO* genes in the B-genome and to strong expression of these genes during fruit ripening. Our analysis also indicated a potential link between the dominant expression of homoeologue genes and the expansion of gene families with fruit ripening and starch metabolism. Previous studies have demonstrated that the B-genome is associated with improved vigour and tolerance to both biotic and abiotic stresses. Consequently, the B-genome is a target for banana breeding programmes^2^. Here, we highlight that the B-genome is of great importance in ethylene biosynthesis and post-harvest banana ripening, which will further our understanding of ripening mechanisms in polyploid fruit.

The *M. balbisiana* genome assembly, along with our previously acquired *M. acuminata* genome data, may aid in the discovery of cultivar-specific sequences that are related to important cultivar-specific traits, including shelf life, quality and stress tolerance. Thus, these resources can be used to build molecular characterization strategies for various cultivars to assist in rapid quality control and the conservation of biodiversity. The data from this study also pave the way for whole-genome association studies, germplasm improvement and genetic modification of bananas to meet increasing commercial demands. These genomic resources and results also reinforce the use of the banana as an appropriate model to study subgenome evolution and fruit biology in triploid variants. Due to the sterility and seedlessness of banana cultivars, further efforts will be needed to leverage the key gene resources from precisely characterized germplasms to achieve effective breeding schemes.

## Methods

### Sample collection

A double haploid of the wild diploid genotype PKW; 2*n* = 2*x* = 22 was provided by the Centre de Coopération Internationale en Recherche Agronomique pour le Développement (CIRAD) for genome sequencing. Fresh, unexpanded leaves were harvested and then frozen immediately with liquid nitrogen to preserve genomic DNA for isolation. High-molecular weight genomic DNA was extracted using a standard cetyltrimethyl ammonium bromide (CTAB) method^62^. DNA integrity was assessed by agarose gel electrophoresis (concentration of agarose gel, 1%; voltage, 150 V; electrophoresis time, 40 min). Finally, DNA was purified from the gel using a QIAquick Gel Extraction kit (QIAGEN).

### Library construction and sequencing

One paired-end and eight mate-pair libraries were constructed for short-read sequencing on the Illumina HiSeq 2000 platform, which generated around 86.34 Gb (166× coverage) of high-quality data. For long-read DNA sequencing, 5.79 million SMRT long reads (58.99 Gb data, 113× coverage) were sequenced using the PacBio Sequel system with libraries of 20-kb insert size; sub-reads had a mean length of 10.2 kb and N50 length of 16.6 kb. One Hi-C library was prepared and sequenced on Illumina NovaSeq 6000 to generate 71.96 Gb (138× coverage) of high-quality data^63^ (Supplementary Table 1). Additional details are available in the Supplementary note.

### Genome assembly

De novo assembly of DH-PKW was performed using wtdbg (v.1.2.8; https://github.com/ruanjue/wtdbg) based on PacBio data (only reads longer than 1 kb were used in assembly). The assembled genome was corrected for two rounds using the ‘wtdbg-cns’ programme in the wtdbg package. We then used the algorithm Arrow (https://github.com/PacificBiosciences/GenomicConsensus), which takes into account all of the underlying data and the raw quality values inherent in SMRT sequencing, to polish the assembly again for the final consensus accuracies. The final consensus contigs were scaffolded using the SSPACE-standard programme^64^ (v.3.0) with meta-pair reads from libraries of insert size 2–20 kb. Based on Hi-C data, 430.02-Mb scaffolds were anchored to 11 pseudo-molecules using LACHESIS software^13^ (Supplementary Fig. 2). Chromosomes were numbered according to the linkage group nomenclature adopted for *M. acuminata*. Additional details regarding genome assembly are provided in the Supplementary note.

### Evaluation of assembly quality

BUSCO (v.3) was used to assess assembly completeness^11^. We mapped 29,610 *M. acuminata*-expressed sequence tags (ESTs) to the assembled genome using BLAT^65^ (v.35) with default parameters. In total, 93.59% of the ESTs were aligned to the genome with identity >90%. Additionally, BWA^66^ v.0.7.12 (aln -l 35) was used to map 59× Illumina reads to the assembly, and 96.11% of the reads were mapped to the assembled genome. Additional details are available in the Supplementary note.

### Genome annotation

Repetitive sequences within the *M. balbisiana* genome were identified by a combination of homology-based and de novo approaches (Supplementary Table 1). Gene structures were annotated iteratively using three main approaches (ab initio predictions, homologue proteins and transcriptome data) that were combined using MAKER^15^ (v.2.31.8) (Supplementary Table 5). Gene functions were annotated according to the best match of the alignments using blastp^67^ (*E*-value < 1 × 10^–5^) against the Swiss-Prot^68^, TrEMBL^68^, NR (https://ftp.ncbi.nlm.nih.gov/blast/db/FASTA/), KOG^69^ and KEGG^70^ databases. Additional details are available in the Supplementary note.

### Transcription factors

We used the iTAK programme^16^ to identify transcription factors based on the consensus rules that are mainly summarized within PlnTFDB and PlantTFDB^71,72^, with families from PlantTFcat^73^ and AtTFDB^74^ used as supporting evidence. In total, we identified 3,329 transcription factor genes in *M. balbisiana* and 3,899 in *M. acuminata* (Supplementary Table 6).

### Gene family analysis

A total of 500,142 genes from 16 plant species with available whole-genome sequences were used for gene family clustering analysis. BLAST^67^ (v.2.2.26; -p blastp) was used to generate pairwise protein sequence alignments with *E*-values of < 1 × 10^–5^. Then, OrthoMCL^22^ was used to cluster similar genes by setting the main inflation value at 1.5 and using default settings for the other parameters. These analyses resulted in 39,358 gene families comprising 393,700 genes from the 16 species (Supplementary Table 7 and Supplementary Fig. 7).

We identified 519 single-copy gene families shared among the 16 species, and constructed a phylogenetic tree using MrBayes (v.3.1.2)^75^ software with the general time-reversible model (Supplementary Fig. 4). Divergence times for the 16 species were also estimated based on fourfold degenerate sites of all single-copy orthologous genes using the MCMCTree programme in the PAML package (v.4.4)^76^ (Supplementary Fig. 5).

CAFÉ (v.2.1)^23^ was used to analyse the expansion and contraction of gene families. A random birth-and-death model was used to assess gene gain or loss in gene families across the specified phylogenetic tree (Supplementary Fig. 9). Families with *P* < 0.05 were considered as significant expansion or contraction, and pathway enrichment analysis of these families was conducted using the enrichment pipeline^77^. Additional details are available in the Supplementary note.

### Whole-genome alignment analysis

MCSCAN^78^ (parameters: -a -e 1e-5 -s 5) was used to detect collinearity within *M. acuminata* (A-genome) and *M. balbisiana* (B-genome) and among various species. Syntenic blocks containing at least ten gene pairs were obtained. All of the orthologous and paralogous gene pairs were extracted from the syntenic blocks for calculation of 4dTv^79^ distances using the HKY substitution model^80^ (Supplementary Fig. 8). Additional details are available in the Supplementary note.

### Orthologous gene pair analysis

BLAST^67^ (v.2.2.26, -p blastp) was used to align *M. acuminata* proteins to *M. balbisiana* proteins for identification of orthologous genes. The value 1 × 10^–5^ was used as a cut-off to define the raw orthologues. We then filtered the BLAST results using two parameters (CIP ≥ 60% and CALP ≥ 60% (ref. ^35^). We identified 25,717 orthologous gene pairs (81.83% consistency with syntenic blocks) between *M. acuminata* and *M. balbisiana* using these two parameters (Supplementary Table 22). The orthologous gene pairs were first aligned using MUSCLE (v.3.8.31)^81^, then the Ka/Ks ratio of each gene pair was calculated using KaKs_Calculator (v.2.0)^82^ with model yn00. The significant difference between Ka/Ks values was detected by Student’s *t*-test.

### Re-sequencing analysis

Nine different genotypes of banana were used for re-sequencing, including the triploid plants BaXiJiao (subgroup *Cavendish*, AAA), Gros_Michel (subgroup *Gros Michel*, AAA), FenJiao (subgroup Pisang Awak, ABB), Kamaramasenge (AAB) and Pelipita (ABB), in addition to the diploid plants Pisang_Mas (subgroup Sucrier, AA), Pisang_Kra (subsp. *malaccensis*, AA), DH-PKW (BB) and balbisiana (BB) (Supplementary Table 13). BaXiJiao, Gros_Michel and FenJiao were obtained from the Tissue Culture Centre of the Chinese Academy of Tropical Agricultural Sciences (CATAS). Pelipita, Pisang_Mas, Pisang_Kra, *M. balbisiana* and Kamaramasenge were provided by the Bioversity International Musa Transit Centre. Genomic DNA was extracted from fresh leaves of seedlings at the five-leaf stage using the CTAB method^62^.

Paired-end reads with libraries of 500-bp insert size were aligned to the A- and B-genomes simultaneously using BWA (v.0.7.12)^66^ with the parameters ‘bwa aln -t 20 -l 35’ (Supplementary Table 14), and only uniquely mapped reads were kept. Potential PCR duplicates were marked using Picard (v.1.54, https://broadinstitute.github.io/picard/) and indexed using the SAMtools package^83^. The Genome Analysis Toolkit^84^ was then used to infer SNPs and InDels. SNP identifications were filtered based on the following parameters: ‘QD < 2.0 || FS > 60.0 || MQ < 40.0 || MQRankSum < −12.5 || ReadPosRankSum < −8.0’, and InDels were filtered based on the following parameters: ‘QD < 2.0 || FS > 200.0 || ReadPosRankSum < −20.0’ (Supplementary Tables 16 and 17). Breakdancer (http://breakdancer.sourceforge.net/)^85^ was used to detect structural variations by checking paired-end reads with an abnormal orientation and/or span. The final structural variations were filtered using the following requirements: (1) minimum size of 100 and maximum size of 1,000,000; (2) minimum score of ≥30; and (3) minimum number of required reads supporting each structural variation ≥5 (Supplementary Table 18). Nucleotide diversity (π) was analysed using VCFtools (v.0.1.13; https://vcftools.github.io/man_latest.html).

### Analysis of homoeologous exchanges

Assessment of read coverage depth was used to detect homoeologous exchanges between the A- and B-subgenomes. We inferred these based on cases where reads coverage of a given region on one parental genome was significantly high while the orthologous one was low or had no coverage. High coverage indicates duplication, and low or no coverage indicates loss^34^. The uniquely mapped paired-end reads were used to calculate the coverage depth of each sample on the A- and B-genomes (Supplementary Figs. 25–27). According to coverage depth, we detected homoeologous exchanges in the triploids ‘FenJiao (ABB)’, ‘Pelipita (ABB)’ and ‘Kamaramasenge (AAB)’ (Supplementary Table 15). Additional details are available in the Supplementary note.

### Transcriptome analysis

Banana fruits at different stages of development (0, 20 and 80 DAF) and ripening (8 and 14 DPH for BX, 3 and 6 DPH for FJ) were obtained from the banana plantation at the Institute of Tropical Bioscience and Biotechnology (Chengmai, Hainan, 20° N, 110° E). Two-month-old BX and FJ banana seedlings were obtained from the Tissue Culture Centre of CATAS and cultured in Hoagland’s solution. Seedlings at the five-leaf stage were treated with 200 mM mannitol for 7 days, 300 mM NaCl for 7 days and low-temperature conditions (4 °C) for 22 h. Fruit, root and leaf samples were frozen in liquid nitrogen and stored at −80 °C until RNA extraction for transcriptome analysis.

Total RNAs were isolated using a plant RNA extraction kit (TIANGEN). Total RNA (3 μg) from each sample was converted to complementary DNA using a RevertAid First-Strand cDNA Synthesis Kit (Fermentas). cDNA libraries were constructed using TruSeq RNA Library Preparation Kit v.2, and were subsequently sequenced on the Illumina HiSeq 2000 platform using the Illumina RNA-Seq protocol. Two biological replicates were used for each sample. A total of 159.14 Gb (Supplementary Table 21) of high-quality clean data were produced. Gene expression levels were calculated as RPKM^86^. Differentially expressed genes were identified by methods previously established with the read count of two replicates for each gene (fold change ≥2; false discovery rate ≤0.001)^87^. Additional details are available in the Supplementary note.

### Weighted gene co-expression network analysis

Gene expression patterns for all identified genes were used to construct a co-expression network using WGCNA (v.1.47)^39^. Genes without expression detected in all tissues were removed before analyses. Soft thresholds were set based on the scale-free topology criterion employed in ref. ^88^. An adjacency matrix was developed using squared Euclidean distance values, and the topological overlap matrix was calculated for unsigned network detection using the Pearson method. Co-expression coefficients >0.55 between the target genes were then selected. Finally, the network connections were visualized using cytoscape^89^.

### Identification of gene families involved in ethylene biosynthesis and starch metabolism pathways

We compared genes related to ethylene biosynthesis and starch metabolism in the *M. balbisiana* genome to those annotated in both *M. acuminata* and other plant genomes, including *B. distachyon*, *O. sativa*, *Arabidopsis thaliana*, *Solanum lycopersicum*, *Prunus persica*, *Populus trichocarpa* and *V. vinifera*. We retrieved protein sequences of these gene families from 16 species (Supplementary Table 51) for homologue-based searches with the criteria similarity >80% and coverage >80%. We then confirmed the presence of the conserved domain within all protein sequences and removed members without a complete domain.

### Determination of total starch content

Banana pulp was immersed in 0.5% sodium bisulfite for 10 min to prevent browning, and then dried at 40 °C for 24 h. Pulp was then ground and centrifuged. The residue was suspended in 5 ml of 80% Ca(NO_3_)_2_ in a boiling water bath for 10 min then centrifuged for 4 min at 4,000 r.p.m. The supernatant was transferred to a 20-ml volumetric flask and the residue was extracted twice with 80% Ca(NO_3_)_2_, which yielded a combined extract volume of 20 ml. All experiments were repeated three times. The total starch content was assessed following methods described by Yang et al.^90^.

### Scanning electron microscopy

Samples were fixed in stubs using double-faced tape and coated with a 10-nm platinum layer in a Bal-tec MEDo020 coating system (Kettleshulme). The prepared samples were analysed using an FEI Quanta 600 FEG scanning electron microscope (FEI Co.). Observations were performed in secondary electron mode while operating at 15 kV.

### Measurement of ethylene production during fruit post-harvest stage

Ethylene production was measured by enclosing fruit samples in an airtight container for 2 h at 25 °C. After incubation, 1 ml of the headspace gas was withdrawn and injected into a gas chromatograph (GC-2010, Shimadzu) fitted with a flame ionization detector and an activated alumina column. Ethylene production measurements were obtained as recommended by the manufacturer.

### Reporting Summary

Further information on research design is available in the Nature Research Reporting Summary linked to this article.

## Supplementary information

Supplementary Information Supplementary Note and Supplementary Figs. 1–27.

Reporting Summary

Supplementary Tables Supplementary Tables 1–52.

## Data Availability

Raw sequence reads for B-genome assembly and transcriptome for all samples were deposited in the CNSA (https://db.cngb.org/search/project/CNP0000292/) of CNGBdb with accession number CNP0000292 and Sequence Read Archive of the National Centre for Biotechnology Information (NCBI) under the BioProject (No. PRJNA432894). Genome assembly and annotation of DH-PKW were submitted to NCBI (No. PYDT00000000). Assembly and gene annotation of the A-genome (DH-Pahang) are available on the Banana Genome Hub (http://banana-genome-hub.southgreen.fr/).
