# Supplementary material for: Musa balbisiana genome reveals subgenome evolution and functional divergence
Source: Nat Plants. 2019 Jul 15;5(8):810–21. doi: 10.1038/s41477-019-0452-6 (PMC6784884; doi:10.1038/s41477-019-0452-6)
Supplement: Supplementary file 1 — Supplementary Note and Supplementary Figs. 1–27. [file 41477_2019_452_MOESM1_ESM.pdf]

nature plants

SUPPLEMENTARY INFORMATION
https://doi.org/10.1038/s41477-019-0452-6

In the format provided by the authors and unedited.

Musa balbisiana genome reveals subgenome evolution and functional divergence

Zhuo Wang[1,12], Hongxia Miao[1,12], Juhua Liu[1,2,12], Biyu Xu[1,12], Xiaoming Yao[3,12], Chunyan Xu[3,12], Shancen Zhao[4], Xiaodong Fang[3], Caihong Jia[1], Jingyi Wang[1], Jianbin Zhang[1], Jingyang Li[2], Yi Xu[2], Jiashui Wang[2], Weihong Ma[2], Zhangyan Wu[3], Lili Yu[3], Yulan Yang[3], Chun Liu[3], Yu Guo[3], Silong Sun[3], Franc-Christophe Baurens[5,6], Guillaume Martin[5,6], Frederic Salmon[6,7], Olivier Garsmeur[5,6], Nabila Yahiaoui[5,6], Catherine Hervouet[5,6], Mathieu Rouard[8], Nathalie Laboureau[9,10], Remy Habas[9,10], Sebastien Ricci[6,7], Ming Peng[1], Anping Guo[1], Jianghui Xie[1], Yin Li[11], Zehong Ding[1], Yan Yan[1], Weiwei Tie[1], Angélique D'Hont[5,6*], Wei Hu[1*] and Zhiqiang Jin[1,2*]

[1]Key Laboratory of Biology and Genetic Resources of Tropical Crops, Institute of Tropical Bioscience and Biotechnology, Chinese Academy of Tropical Agricultural Sciences, Haikou, China. [2]Key Laboratory of Genetic Improvement of Bananas, Hainan province, Haikou Experimental Station, China Academy of Tropical Agricultural Sciences, Haikou, China. [3]BGI Genomics, BGI-Shenzhen, Shenzhen, China. [4]BGI Institute of Applied Agriculture, BGI-Shenzhen, Shenzhen, China. [5]CIRAD, UMR AGAP, Montpellier, France. [6]AGAP, Univ Montpellier, CIRAD, INRA, Montpellier SupAgro, Montpellier, France. [7]CIRAD, UMR AGAP, Guadeloupe, France. [8]Bioversity International, Montpellier, France. [9]CIRAD, UMR BGPI, Montpellier, France. [10]BGPI, CIRAD, INRA, Montpellier SupAgro, Montpellier, France. [11]Waksman Institute of Microbiology, Rutgers, The State University of New Jersey, Piscataway, NJ, USA. [12]These authors contributed equally: Zhuo Wang, Hongxia Miao, Juhua Liu, Biyu Xu, Xiaoming Yao, Chunyan Xu. *e-mail: dhont@cirad.fr; huwei2010916@126.com; 18689846976@163.com

Supplementary Information

1. Genome assembly

1.1 Sample collection

A double haploid (DH) of the wild diploid genotype Pisang Klutuk Wulung (PKW; 2n=2x=22) was provided by the centre de coopération internationale en recherche agronomique pour le développement (CIRAD) for genome sequencing. Pisang Klutuk Wulung (PKW) is a wild *Musa balbisiana* accession collected in 1985 in Indonesia and conserved in the field collection of the CRB Plantes Tropicales Antilles CIRAD-INRA Guadeloupe (French West Indies) under accession code PT-BA-00302 and as *in vitro* plantlets at the Bioversity's International Transit Center (ITC) hosted by the Katholieke Universiteit Leuven in Belgium, under the accession code ITC1587. The DH-PKW was obtained in Guadeloupe through anther culture and spontaneous chromosomes doubling[10,91] and is conserved in the CIRAD field collection in Guadeloupe. Its homozygous status was verified with SSR markers and endogenous Banana Streak Virus PCR based genotyping[92].

Plants were grown in a greenhouse where the minimum and maximum temperatures were 25°C and 30°C, respectively. Fresh unexpanded leaves were harvested, and then frozen immediately with liquid nitrogen in order to preserve genomic DNA for isolation. High molecular weight genomic DNA was extracted using a standard cetyltrimethyl ammonium bromide (CTAB) method[62]. DNA integrity was assessed by agarose gel electrophoresis (concentration of agarose gel: 1%,

voltage: 150 V, electrophoresis time: 40 min). Finally, DNA was purified from the gel using a QIAquick Gel Extraction kit (QIAGEN, Shanghai, China).

1.2 Library construction and sequencing

DNA was extracted from DH-PKW leaves using a standard CTAB extraction[62]. We used a whole genome shotgun strategy and next-generation sequencing based on Illumina HiSeq 2000 platform. In order to reduce the risk of non-randomness, we constructed one paired-end and eight mate-pair libraries with insert sizes of 500 bp, 2 kb, 5 kb, 10 kb, and 20 kb. To reduce the effect of sequencing error, we stringently filtered reads by removing reads meeting the following criteria:

Type (1): Reads with ≥ 10% and ≥ 3% unidentified nucleotides for short and long insert size libraries, respectively.

Type (2): Reads from short-insert libraries having more than 40% of bases with quality score less than 7, and reads from long-insert libraries that contained more than 20% bases with quality scores less than 7.

Type (3): Reads with > 10 bp aligned to the adapter sequence, while allowing ≤ 2 bp mismatches.

Type (4): Small paired-end reads in short-insert libraries that overlapped by more than 10 bp with the corresponding paired end.

Type (5): Read 1 and read 2 of two paired-end reads that were completely identical (considered to be PCR duplication products).

Following the above quality control and filtering steps, 86.34 Gb (166x coverage) of sequencing data was retained for assembly (Supplementary Table 1).

For PacBio sequencing, SMRT libraries were constructed using the PacBio 20-kb protocol (https://www.pacb.com/). Six SMRT cells were loaded and 5.79 million long reads were produced of 20-kb insert size libraries. A total of 58.99 Gb data was generated by PacBio Sequel system (Supplementary Table 1). The subreads have a mean length of 10.2 kb and N50 length of 16.6 kb, with 98.83% of the raw data with length > 1 kb.

For Hi-C sequencing, leaves of HD-PKW were collected and cut into 5mm × 5mm pieces. It was soaked into 18% formaldehyde for 5 minutes, then soaked into 2% formaldehyde for 30 minutes, and kept 30 minutes on ice after adding 2M glycine solution. Finally rinsed twice with sterile distilled water and frozen into liquid Nitrogen. Hi-C library were prepared using the method described by Lieberman-Aiden et al.[63]. We constructed a Hi-C library with DNA fragment from 300 to 700bp and sequenced on Illumina NovaSeq 6000 platform. After filtering adapter contamination and low quality reads, we got 71.96 Gb (138 × coverage) clean data (Supplementary Table 1).

1.3 Estimation of genome size using k-mer analysis

K-mers are artificial sequences with K nucleotide length. A raw sequence read with L bp contains (L-K+1) K-mers, if the length of each K-mer is K bp. The frequency of each K-mer can be calculated from the sequence reads. Typically, K-mer frequencies that are plotted against the sequence depth follow a Poisson distribution in any given dataset. However, sequencing errors may lead to higher representation of K-mers at low frequencies. The genome size can be calculated from the formula

G=K_num/K_depth, where K_num is the total number of K-mers, and K_depth denotes the K-mer frequency occurring more than other frequencies. Here, we used a K of 17 and K_num of 25,507,921,320, and K_depth of 49. We thus estimated the genome size to be 520.57 Mb (Supplementary Fig. 1).

1.4 Genome assembly

De novo assembly of the B-genome was performed using wtdbg (version 1.2.8, https://github.com/ruanjue/wtdbg) based on ~ 113× PacBio data (only reads longer than 1 kb were used in the assembly), the following parameters were used: -t 20 --tidy-reads 5000 -k 0 -p 19 -S 1 --rescue-low-cov-edges. The assembled genome was first corrected for two rounds using "wtdbg-cns" program implemented in wtdbg package. Then we used algorithm Arrow (https://github.com/PacificBiosciences/GenomicConsensus), which takes into account all of the underlying data and the raw quality values inherent in SMRT sequencing, to polish the assembly again for the final consensus accuracies. Further scaffolding was performed by SSPACE v3.0 program[64] using meta-pair reads from libraries of 2 kb to 20 kb insert-size. The total scaffold size was 492.76 Mb and the N50 (50% of the genome in fragments of this length or longer) was 5.05 Mb. The total contig size was 491.47 Mb, and the N50 was 1.83 Mb (Supplementary Table 2).

The quality and completeness of our assemblies were assessed in several ways. BUSCO[11] (version 3) was used to assess assembly completeness by mapping 1,440 conserved plant orthologous genes to the assembled genome and 1,312 (91.1%) can be completely found in our assembly. Then, 29,610 banana-expressed sequence tags

(ESTs) available in the NCBI dbEST database (http://www.ncbi.nlm.nih.gov/dbEST/) were used to map the assembled genome using BLAT[65], and 93.59% were aligned to the assembly with at least 90% identity. Additionally, 59 × Illumina reads generated from the 500 bp insert size libraries were mapped onto the assembly using BWA[66] (version 0.7.12, parameters: aln -l 35), and 96.11% of the data could be mapped on the assembly.

1.5 Pseudomolecules construction

Hi-C technology enables the generation of genome-wide 3D proximity maps and is an efficient strategy for sequences cluster, ordered, and orientation for pseudomolecule construction[13]. The 138.38 × clean Hi-C reads were first truncated at the putative Hi-C junctions and then the resulting trimmed reads were aligned to the assembly with BWA aligner[66] (version 0.7.12) with default parameters. 94.54% of the trimmed reads were mapped on the assembly. Only uniquely aligned pairs reads whose mapping quality more than 20 were remained for further analysis. Invalid read pairs, including Dangling-End and Self-cycle, Re-ligation and Dumped products, were filtered by HiC-Pro (version 2.8.1)[12]. Finally, we got 169.7 Mb (70.61%) uniquely mapped read pairs. Then scaffolds were cut into 200 kb windows for correcting the potential scaffolding error using Hi-C valid read pairs. 84.82% of uniquely mapped read pairs were valid interaction pairs and they were used for clustered, ordered and orientated scaffolds onto chromosomes by LACHESIS software[13] with the following parameters:

CLUSTER_MIN_RE_SITES = 73;

CLUSTER_MAX_LINK_DENSITY=3；

CLUSTER_NONINFORMATIVE_RATIO = 1.5;

ORDER_MIN_N_RES_IN_TRUN=15;

ORDER_MIN_N_RES_IN_SHREDS=15.

Finally, 294 scaffolds (total size was 430.02 Mb) were anchored on 11 pseudomolecules (Supplementary Fig. 2 and Supplementary Table 3). The chromosomes were named according to the linkage group nomenclature adopted in *M. acuminata*.

2. Genome annotation

2.1 Repeat annotation

Transposable elements (TE) in *M. balbisiana* were identified by a combination of homology-based and *de novo* approaches. Firstly, the homology-based approach with RepeatMasker (version 4.0.6)[93] and RepeatProteinMask was used to search Repbase (release 21.01)[94], a database of known DNA/protein TEs. Secondly, an *ab initio* repeat library, which combined Piler (version 1.0)[95], RepeatScout (version 1.0.5)[96], and LTR-FINDER (version 1.0.5)[97], were employed to build the *de novo* repeat library of B-genome. Then we used RepeatMasker[93] (Version 4.0.6) to identify repeat elements based on the *de novo* repeat database. The tandem repeats were annotated using Tandem Repeats Finder (version 4.09)[98]. Lastly, the redundancy between the two methods was eliminated in order to generate combined data (Supplementary Table 4). The most abundant repeat grouping was the Long Terminal Repeat retroelements (LTR), which represented 46.06% of the genome. LINEs were quite underrepresented,

and totally just 1.30% of the genome. DNA transposable elements constituted 2.12% of the B-genome. In addition, 4.94% of the genome was classified as repetitive, but could not be further characterized. The same approaches and parameters were used to annotate TEs in *M. acuminata* (A-genome) (Supplementary Table 4).

To infer the insertion time of LTR retrotransposon, full-length LTR retrotransposons were identified using LTRharvest[99] and LTRdigest[100] included in Genome Tools (version 1.5.8) analysis system[101]. Timing of insertion was based on the divergence of the 5’ and 3’ LTR sequences of each copy[102]. The 5’ and 3’ LTRs were aligned using MUSCLE (version 3.8.31)[81], and the substitutions per nucleotide site were calculated by a custom script. The insertion time was estimated using an average base substitution rate of 1.3E-8[103]. The timing of insertion indicates a very recent wave of LTR retrotransposon amplification (the highest peak at 0-0.5 MYA) in *M. balbisiana* (Supplementary Fig. 3).

2.2 Gene structure annotation

Identification of protein-coding genes involved homolog-based prediction, *de novo* predictions, and the use of RNA-Seq data as follows.

(1) Homolog-based prediction. Homologous proteins of *M. acuminata* (DH Pahang v2), *A. thaliana* (TAIR10), *Z. mays* (B73, v4), *B. distachyon* (v3.0) and S. *bicolor* (Ensembl release-41) were aligned to the B-genome using TblastN with an E-value cutoff of 1e-5. The aligned sequences, and their corresponding query proteins were then filtered and passed to Exonerate (version 2.2.0, parameters: --model protein2genome -percent 20 -minintron 10, -maxintron 50000)[104] to search for

accurate spliced alignments.

(2) *De novo* gene prediction. *De novo* prediction was performed on the transposons-masked genome. Augustus (version 3.2.1)[105] and SNAP (version 2006-07-28)[106] with training model parameters of B-genome were used to predict coding genes.

(3) RNA-Seq assist prediction. Six transcriptome data from *M. balbisiana* were sequenced. The RNA-seq reads were mapped to B-genome using HISAT2 (version 2.0.1-beta; parameters: -max-intronlen 160000 -no-discordant -no-mixed)[107], and the alignments results were assembled by StringTie (version 1.2.1)[108] with default parameters to obtain the reference-based gene structures. In order to get the more perfect alignments, the splice sites were validated and transcripts were assembled again into gene structures by PASA_lite software (https://github.com/PASApipeline/PASA_Lite).

(4) Integration of final consensus gene set. Final integrated gene models were derived from MAKER[15] (version 3.31.8) with upper Augustus and SNAP *de novo* prediction, five protein-based homolog predicted gene structures, and RNA-Seq based transcripts structures. Finally, the *M. balbisiana* gene set contains 35,148 genes with an average gene length of 5 kb (Supplementary Table 5).

Genome annotation completeness was assessed using BUSCO v3[11] with the embryophyta database of 1,440 single copy orthologs, and 94% (1,348) of orthologous genes are completely found in our gene sets.

2.3 Gene function annotation

Gene functions were annotated according to the best match of the alignments using BLAST (version 2.2.26, parameters: -p blastp, -e 1e-05 -b 5 -v 5)[67] against the Swiss-Prot[68], TrEMBL (Uniprot release 2018_07)[68], KOG (release 20090331)[69] and NR database (release 20170924). Protein motifs and domains were determined by InterProScan (version 5.16)[109] against publicly available databases such as PANTHER (http://www.pantherdb.org/), Gene3D[110], SUPERFAMILY[111], Pfam[112], SMART[113], and PROSITE[114]. Gene Ontology[115] functional information was retrieved from NR by converting NR accession ID to GO terms. We also mapped all proteins to KEGG orthologs (Release 87)[70] using balstp (-e 1e-5 -b 5 -v 5) to find the best hit for each gene. Totally, 92% of the genes had assigned function annotation.

2.4 ncRNA annotation

Four types of non-coding RNAs were detected in the whole genome. tRNAs were predicted by tRNAscan-SE (version 1.23) [116] with eukaryote parameters. The miRNAs and snRNAs were predicted using INFERNAL[117] software by searching against the Rfam database (Release 12.0)[118]. rRNAs were identified by aligning to the template rRNA (5S, 5.8S, 18S rRNA from *Arabidopsis thaliana* and 28S from rice) to assembled genome using blastn (version 2.2.26)[67] with *E*-value <1e-5. The annotation predicted 9,134 non-coding RNAs (Supplementary Table 52).

3. Genome evolution

3.1 Genome data used in evolutionary analysis

The gene sets of the fifteen species were downloaded: *A. thaliana* (TAIR10), *B. distachyon* (v3.1), *A. officinalis* (v1.1), A. comosus (JGI_v13), *E. guineensis* (v5), *M.*

acuminata (DH Pahang v2), *O. sativa* (IRGSP-1.0), *P. trichocarpa* (JGI_v13), *S. bicolor* (JGI_v13), *Z. mays* (B73, v4), *P. equestris* (NCBI), *S. polyrhiza* (JGI_v13), *V. vinifera* (Genoscope), *S. lycopersicum* (ITAG3.2) and *A. trichopoda* (AMTR1.0). The gene sets were used for gene clustering, phylogenetic reconstructions, divergence time estimations, and identification of chromosome collinearity, among other analyses. All gene sets were processed and filtered using the following criteria:

1) Removal of genes when internal stop codons were present in the CDS.

2) Genes were retained with the longest alternative splicing sites.

3) Mixed bases were recoded to NNN for the codon, and the corresponding protein was coded to X.

3.2 Gene clustering by OrthoMCL

In total, 500,142 genes from above plants were used for gene family clustering analysis. First, blastp[67] all-by-all (version 2.2.26) was used to generate pairwise protein sequence alignments with E-value less than 1e-5. Second, OrthoMCL (Version 1.4) [22] was used to cluster similar genes by setting the main inflation value at 1.5 and using the default settings for other parameters. In total, 39,358 gene families comprising 393,700 genes from nine species were generated (Supplementary Table 7 and Supplementary Figs. 7-8).

3.3 Phylogenetic analyses

The 519 single-copy orthologous genes shared for the sixteen species were used to construct a phylogenetic tree. The protein sequence from all single-copy orthologous genes were aligned using MUSCLE[81]. The alignments were then changed

to nucleotide sequence using each gene's corresponding CDS sequence. Each amino acid was substituted to the corresponding triplet bases from its CDS according to the same ID information using a custom Perl script, and for the gap (-) in protein alignment, one gap (-) will be substituted into 3 gaps (---). We extracted four-fold degenerate (4d) sites and phase 1 sites of all single-copy orthologous genes in each species, and concatenated them to one super-gene for phylogeny construction separately. We constructed a phylogenetic tree using MrBayes (version 3.1.2)[75] software with GTR model (Supplementary Fig. 3).

We further estimated the divergence time for sixteen species based on 4 d sites of all single-copy orthologous genes. Markov chain Monte Carlo algorithm for Bayes estimation was adopted to estimate the neutral evolutionary rate and species divergence time using the program MCMC Tree with JC69 model of the PAML package[76]. The following constraints were used for time calibrations: (i) the *O. sativa* and *B. distachyon* divergence time (40-53 million years ago (MYA))[119]; (ii) the *P. trichocarpa* and *A. thaliana* divergence time (100-120 MYA)[120]; and (iii) 200 MYA as the upper boundary for the earliest-diverging angiosperms[121]. The estimated divergence time between *M. acuminata* and *M. balbisiana* was 5.4 MYA (1.8-13.3 MYA) (Supplementary Fig. 5).

3.4 Expansion and contraction of gene families

Based on the identified gene families and the constructed phylogenetic tree with predicted divergence time of the 16 species, we used CAFÉ software (v2.1)[23] to analyze gene families' expansion and contraction (Supplementary Fig. 9). First,

families with too much change in size were discarded (families with gene number ⩾ 200 in one species and ≤ 2 in all other species), then families with most recent common ancestor (MRCA) size equal to 0 predicted by parsimony method were also filtered.

In CAFÉ, a random birth and death model is proposed to study gene gain or loss in gene families across a specified phylogenetic tree. Branch length values represented the divergence time. The global parameter λ (lambda), which describe both the gene birth (λ) and death (μ = -λ) rate across all branches in tree for all gene families was estimated using maximum likelihood method. Then, conditional p-value was calculated for each gene family, and family with conditional *p*-value less than 0.01 was considered to have an accelerated rate for gene gain or loss.

Finally, we predicted a total of 11,499 MRCA families. There are 1,761 gene families that expanded in A-genome and 392 expanded in B genome. We analyzed if they are tandem duplication in one genome or gene loss in another genome. We first checked the 1,761 families expanded in A-genome in detail, and we found: (1) 245 families contain tandem duplication genes (totally contain 776 tandem duplication genes; criteria of tandem duplication: *e*-values < 1e-20 and identity > 40%, with a maximum of five intervening genes); (2) 360 gene families are contraction (gene loss) in B-genome compared with their common ancestor; (3) except for the 360 families, the rest of the 1,401 families are no size change in B-genome. Among the rest of 1,401 families, 1,255 families in A-genome have one more gene than B-genome, and 143 families have two more genes than B genomes. Totally, 14% of the expansion

families in A-genome have tandem duplication, and 20% families are gene loss in B-genome. And among the 1,401 families, 99.9% (1,398) has one or two more genes than B genome. The total statistics of the 1,761 expanded families in *M. acuminata* and 392 expanded families in *M. balbisiana* was summarized in Supplementary Tables 8-9.

To further analyze the similarity of those genes in the 1,401 families (contain 4,740 genes) that expanded in A-genome but no size change in B-genome and the 332 families (contain 1,202 genes) that expanded in B-genome but no size change in A-genome, we did all-versus-all blastp (E value < 1e-5) alignment of all those genes from A- and B-genome. Based on the blast results, we calculated the CIP value (Cumulative Identity Percentage - Sum of all HSPs' identity sequence divided by the cumulative aligned length)[35,36] of each gene pair to evaluate the sequence similarity. For genes in each same family, we calculated three groups of CIP: (1) CIP of all gene-pairs in A-genome, (2) CIP of all gene-pairs in B-genome, and (3) CIP of all gene-pairs between A- and B-genomes. Then, we calculated the average CIP of the upper three sets. Finally, for 1,401 families (expanded in A-genome but no size change in B-genome), the average CIP of gene-pairs in A-genome (CIP_A), B-genome (CIP_B) and between A- and B-genomes (CIP_A vs B) are 71.76, 70.13 and 76.97, respectively. For 332 families (expanded in B-genome but no size change in A-genome), the average CIP of CIP_A, CIP_B and CIP_A vs B are 71.14, 68.32 and 75.58, respectively (Supplementary Tables 8-9). According to this result, the genes' similarity between A- and B-genomes is higher than that in themselves.

The significantly expanded gene families in B-genome (p-value ≤ 0.05) were mapped to KEGG pathways[70] for further functional enrichment analysis (Supplementary Table 10). The KEGG pathway enrichment analysis was conducted using the enrichment methods[77], which implemented hypergeometric test algorithms and the Q-value (FDR, False Discovery Rate) was calculated to adjust the p-value using R package (https://github.com/StoreyLab/qvalue).

3.5 Genome duplication analysis

The all-versus-all blastp[67] method (version 2.2.26, E-value<1e-5) was used to detect paralogous genes in *M. acuminata*, *M. balbisiana* and *A. thaliana* as well as orthologous genes in *M. acuminata-M. balbisiana*, *M. acuminata-A. thaliana* and *M. balbisiana-A. thaliana.* Syntenic blocks were detected using MCSCAN (parameters: -a -e 1e-5 -s 5) [78]. We extracted all the paralogous and orthologous gene pairs from syntenic blocks in those species to further calculate the 4dTv[79] distances using the HKY substitution model[80]. The distribution of 4dTv (Supplementary Fig. 6) confirmed the banana shared recent and ancient WGD.

4. Analysis of homoeologous exchanges

Assessment of read coverage depth was used to detect homoeologous exchanges (HEs) between A- and B- subgenome[34]. We detected the HEs in three triploids FenJiao (ABB), Pelipita (ABB), and Kamaramasenge (AAB). The uniquely mapped Illumina paired-end reads (Supplementary Table 14) were used to calculate the coverage depth of each samples on A- and B-genome (Supplementary Figs. 25-27). Suppose "A-Cov" represents the coverage peak on A-genome and "B-Cov" represents

the coverage peaks on B-genome of three triploids. For example, “A-Cov” and “B-Cov” of FenJiao were 8 and 19 respectively (Supplementary Fig. 25). We calculated the average depth on each 10 kb windows. For ABB group, windows with depth >= “A-Cov + B-Cov” in B-genome and depth >= “A-Cov+B-Cov/2” in A-genome were considered as duplicated windows. For AAB group, the same principle (B-genome depth>= “A-Cov/2 + B-Cov”; A-genome depth >= “A-Cov+B-Cov”) was used to detect the duplicated window. Adjacent duplicated windows that were at most 5 windows distant were linked together. Only regions spanning more than 8 windows (80 kb) were retained. Totally, we initially identified 263 regions which coverage depth was high than the corresponding threshold on one parents. Then, based on the homoeologous regions on chromosomes that were defined by syntenic blocks, we confirmed a total of 161 segments where the orthologous region can be found in the other parental genome with at least 50% orthologous gene pairs existing in syntenic blocks. We found Chr10 of B-genome in Kamaramasenge and Chr02, Chr07 and Chr11 of A-genome in Pelipita were almost entirely replaced by the corresponding homoeologous chromosomes (Fig. 2). Among the 161 segments, 91 are located on these four chromosomes. Excluding these 4 chromosomes, we identified 48 segmental HEs in FenJiao (ABB), 18 in Pelipita (ABB) and 4 in Kamaramasenge (AAB) (Supplementary Table 15).

5. Transcriptome analysis

5.1 Plant materials and treatments

Two cultivated varieties of BaXiJiao (*Musa acuminata* L. AAA group cv. BaXi

Jiao; hereafter referred to as BX) and FenJiao (*Musa* ABB Pisang Awak, ITC0213; hereafter referred to as FJ) were used for transcriptomic analysis. Banana fruits at different stages of development, including at 0 days after flower (DAF), 20 DAF, and 80 DAF (0 day post-harvest: 0 DPH), were obtained from the banana plantation at the Institute of Tropical Bioscience and Biotechnology (Chengmai, Hainan, 20 N, 110 E). The degree of ripening in the postharvest ripening process can be divided into the following seven stages according to Pua et al.[122]: full green (FG), trace yellow (TY), more green than yellow (MG), more yellow than green (MY), green tip (GT), full yellow (FY), and yellow flecked with brown spots (YB). Fruits at 8 and 14 DPH in BX reached MG and FY stages, respectively, whereas those of 3 and 6 DPH in FJ reached MG and FY stages, respectively. The fruit samples (0 DAF, 20 DAF, 80 DAF, 8 DPH and 14 DPH for BX; 0 DAF, 20 DAF, 80 DAF, 3 DPH and 6 DPH for FJ) were frozen in liquid nitrogen and stored at -80°C until RNA extraction was conducted for transcriptome analysis. Two-month-old banana seedlings of BX and FJ were obtained from the Tissue Culture Center of CATAS. Banana seedlings at five leaves stage were treated with 200 mM mannitol for 7 days, 300 mM NaCl for 7 days, and low temperature conditions (4°C) for 22 hours. The leaves were sampled for transcriptome analysis. The leaves and roots sampled from banana seedlings at five leaves stage cultured in Hoagland’s solution were used as control.

5.2 RNA-Seq sequencing and expression analysis

Total RNA was isolated using a plant RNA extraction kit (TIANGEN, Beijing,

China). Three μg of total RNA from each sample was converted to cDNA using a RevertAid First-Strand cDNA Synthesis Kit (Fermentas, Beijing, China). cDNA libraries were constructed using TruSeq RNA Library Preparation Kit v2, and were subsequently sequenced on the Illumina HiSeq 2000 platform using the Illumina RNA-seq protocol. Two biological replicates were used for each sample.

Paired end reads with 90-bp were produced on HiSeq 2000 platform of all samples. A total of 159.14 Gb of high-quality clean data was produced (Supplementary Table 21) and aligned using SOAPaligner/SOAP2 version 2.21 with parameters "-m 0 -x 1000 -s 40 -l 32 -v 5 -r 1 -p 3"[123]. Clean reads of FJ samples were simultaneously aligned to the A- and B-genome, and clean reads of BX samples were mapped to A-genome (*M. acuminata*). Gene expression levels were calculated as RPKM[76]. Differentially expressed genes were identified by the methods established by Audic et al. (1997) with the read count of two replicates for each gene (fold change ≥ 2; FDR ≤ 0.001)[87]. For homoeolog gene pairs, the genes that dominantly expressed in A-subgenome must meet: (1) the genes in A-subgenome showed upregulation (Log2 based RPKM>1) at least in 6 samples relative to their homoeologs in B-subgenome; (2) their homoeologs in B-subgenome did not show upregulation (Log2 based RPKM>1) relative to the genes in A-subgenome in the rest of samples. The genes that dominantly expressed in B-subgenome must meet: (1) the genes in B-subgenome showed upregulation (Log2 based RPKM > 1) at least in 6 samples relative to their homoeologs in A-subgenome; (2) their homoeologs in A-subgenome did not show upregulation (Log2 based RPKM > 1) relative to the genes in

B-subgenome in the rest of samples.

6. References

91. Bakry, F., Assani, A., & Kerbellec, F. Haploid induction: androgenesis in *Musa balbisiana*. *Fruit* **63**, 45-49 (2008).

92. Umber, M. et al. Marker-assisted breeding of *Musa balbisiana* genitors devoid of infectious endogenous Banana streak virus sequences. *Mol. Breeding* **36**, 74 (2016).

93. Chen, N. Using RepeatMasker to identify repetitive elements in genomic sequences. *Curr. Protoc. Bioinformatics* **25**, 4-10 (2004).

94. Jurka, J. et al. Repbase Update, a database of eukaryotic repetitive elements. *Cytogenet. Genome Res.* **110**, 462-467 (2005).

95. Edgar, R. C., & Myers, E. W. PILER: identification and classification of genomic repeats. *Bioinformatics* **21**, i152-i158 (2005).

96. Price, A. L., Jones, N. C., & Pevzner, P. A. *De novo* identification of repeat families in large genomes. *Bioinformatics* **21**, i351-i358 (2005).

97. Xu, Z., & Wang, H. LTR_FINDER: an efficient tool for the prediction of full-length LTR retrotransposons. *Nucleic Acids Res.* **35**, W265-W268 (2007).

98. Benson, G. Tandem repeats finder: a program to analyze DNA sequences. *Nucleic Acids Res.* **27**, 573 (1999).

99. Ellinghaus, D., Kurtz, S., & Willhoeft, U. LTRharvest, an efficient and flexible software for *de novo* detection of LTR retrotransposons. *BMC Bioinformatics*

9, 18 (2008).

100. Steinbiss, S. et al. Fine-grained annotation and classification of de novo predicted LTR retrotransposons. *Nucleic Acids Res.* **37**, 7002-7013 (2009).

101. Gremme, G., Steinbiss, S., & Kurtz, S. GenomeTools: a comprehensive software library for efficient processing of structured genome annotations. *IEEE/ACM Transactions on Computational Biology and Bioinformatics (TCBB)* **10**, 645-656 (2013).

102. SanMiguel, P. et al. Nested retrotransposons in the intergenic regions of the maize genome. *Science* **274**, 765-768 (1996).

103. Ma, J., & Bennetzen, J. L. Rapid recent growth and divergence of rice nuclear genomes. *Proc. Natl. Acad. Sci. USA* **101**, 12404-12410 (2004).

104. Slater, G. S. C., & Birney, E. Automated generation of heuristics for biological sequence comparison. *BMC Bioinformatics* **6**, 31 (2005).

105. Stanke, M. et al. AUGUSTUS: ab initio prediction of alternative transcripts. *Nucleic Acids Res.* **34**, W435-W439 (2006).

106. Korf, I. Gene finding in novel genomes. *BMC bioinformatics* **5**, 59 (2004).

107. Kim, D., Langmead, B., & Salzberg, S. L. HISAT: a fast spliced aligner with low memory requirements. *Nature Methods* **12**, 357 (2015).

108. Pertea, M. et al. StringTie enables improved reconstruction of a transcriptome from RNA-seq reads. *Nature biotechnol.* **33**, 290 (2015).

109. Zdobnov, E. M., & Apweiler, R. InterProScan–an integration platform for the signature-recognition methods in InterPro. *Bioinformatics* **17**, 847-848 (2001).

110. Yeats, C. et al. Gene3D: modelling protein structure, function and evolution. *Nucleic Acids Res.* **34**, D281-D284 (2006).

111. Gough, J. The SUPERFAMILY database in structural genomics. *Acta Crystallographica Section D: Biological Crystallography* **58**, 1897-1900 (2002).

112. Mistry, J., & Finn, R. Pfam: a domain-centric method for analyzing proteins and proteomes. *Comparative Genomics* **396**, 43-58 (2007).

113. Schultz, J. et al. SMART, a simple modular architecture research tool: identification of signaling domains. *Proc. Natl. Acad. Sci. USA* **95**, 5857-5864 (1998).

114. Hulo, N. et al. The PROSITE database. *Nucleic Acids Res.* **34**, D227-D230 (2006).

115. Ashburner, M. et al. Gene ontology: tool for the unification of biology. The Gene Ontology Consortium. *Nat. Genet.* **25**, 25-29 (2000).

116. Lowe, T. M., & Eddy, S. R. tRNAscan-SE: a program for improved detection of transfer RNA genes in genomic sequence. *Nucleic Acids Res.* **25**, 955-964 (1997).

117. Nawrocki, E. P., Kolbe, D. L., & Eddy, S. R. Infernal 1.0: inference of RNA alignments. *Bioinformatics* **25**, 1335-1337 (2009).

118. Griffiths-Jones, S. et al. Rfam: an RNA family database. *Nucleic Acids Res.* **31**, 439-441 (2003).

119. International Brachypodium Initiative. Genome sequencing and analysis of the model grass *Brachypodium distachyon*. *Nature* **463**, 763 (2010).

120. Tuskan, G. A et al. The genome of black cottonwood, *Populus trichocarpa*

(Torr. & Gray). *Science* **313**, 1596-1604 (2006).

121. Magallón, S., Hilu, K. W., & Quandt, D. Land plant evolutionary timeline: gene effects are secondary to fossil constraints in relaxed clock estimation of age and substitution rates. *J. Exp. Bot.* **100**, 556-573 (2013).

122. Pua, E. C. et al. Malate synthase gene expression during fruit ripening of Cavendish banana (*Musa acuminata* cv. Williams). *J. Exp. Bot.* **54**, 309-316 (2003).

123. Li, R. et al. SOAP: short oligonucleotide alignment program. *Bioinformatics* **24**, 713-714 (2008).

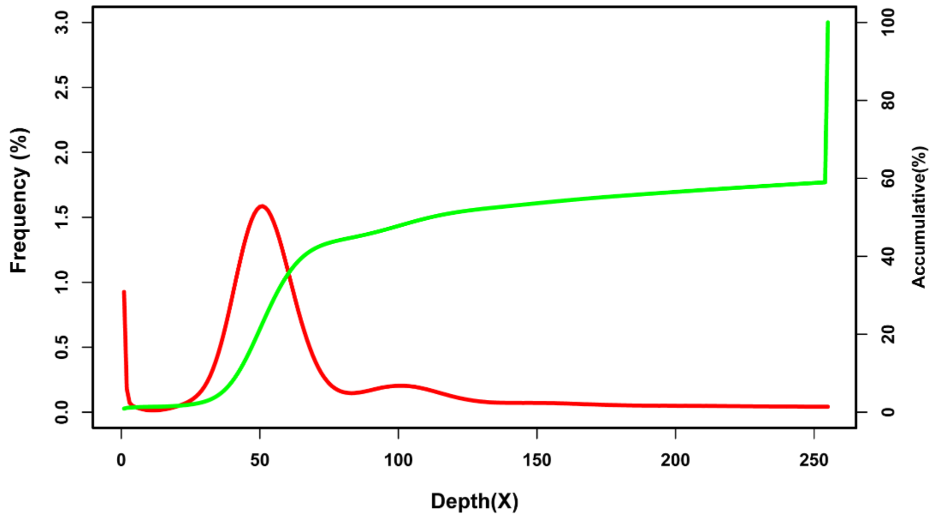

k-mer	k-mer number	peak depth	genome size(bp)	used bases	used reads	unique k-mer
17	25,507,921,320	49	520,569,822	30,366,573,000	303,665,730	271,977,289

Supplementary Figure 1. The K-mer analysis used to estimate B-genome size.

The frequency of 17-mers are shown representing 17 bp sequences within reads (after filtering) from the clean reads of short-insert size libraries. The red curve shows the K-mer frequency distribution, and the green curve shows the cumulative distribution of K-mer frequency. Genome size is estimated as: (total K-mer number) / (the peak depth). The estimate for genome size was 520.57 Mb.

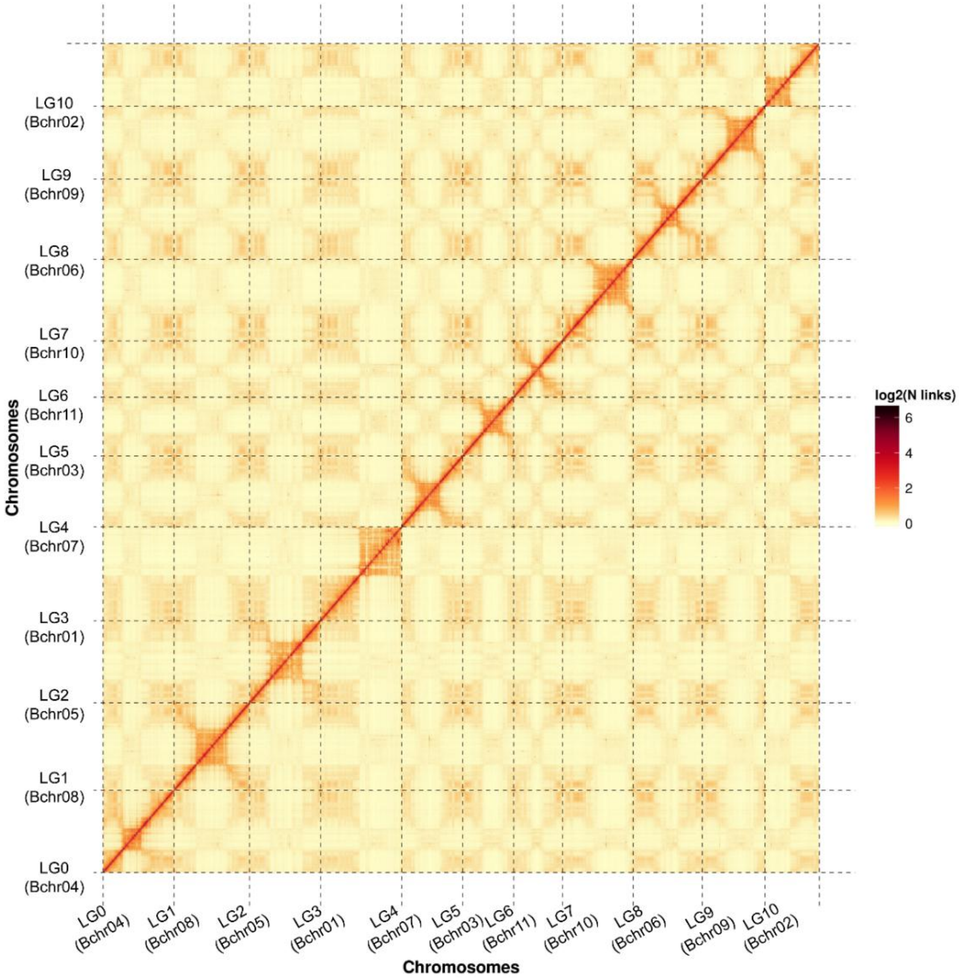

Supplementary Figure 2. The Hi-C chromatin interaction map for the 11 pseudomolecules of B-genome.

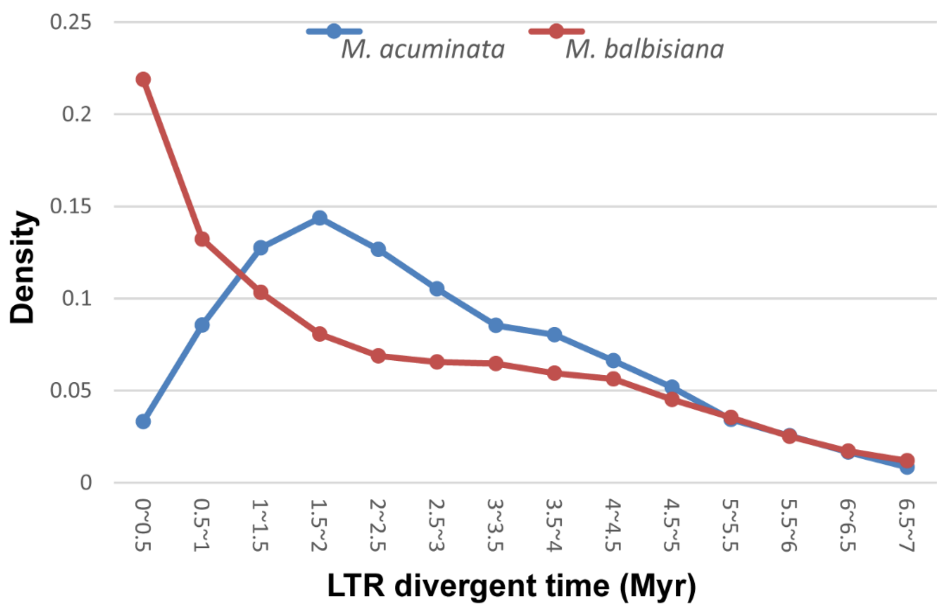

Supplementary Figure 3. Timing of LTR retrotransposon insertions. The blue line represents the LTR insertion time (million years ago) of A-genome, while the red line represents the insertion time of B-genome.

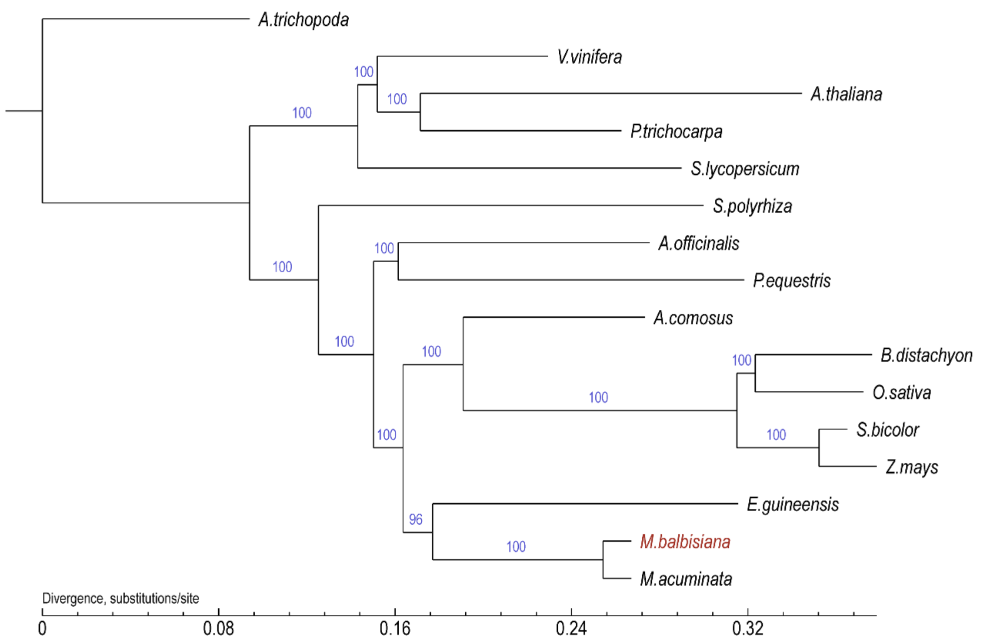

Supplementary Figure 4. Phylogenetic tree on the basis of single-copy orthologous genes shared among *M. acuminata*, *M. balbisiana* and 14 other plant species.

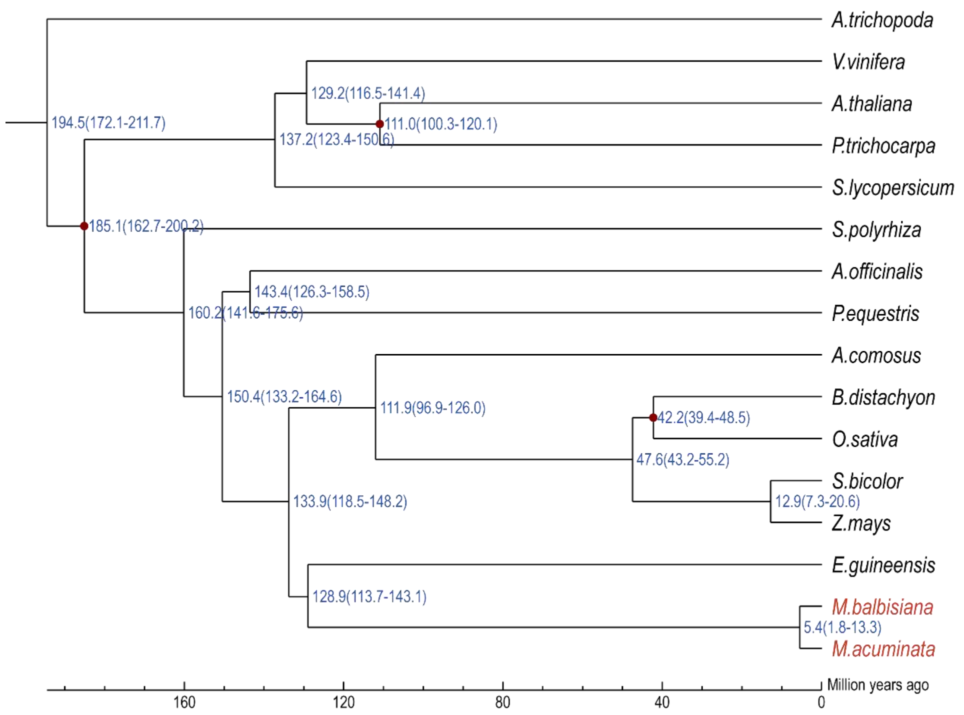

Supplementary Figure 5. Estimation of the divergence time of the B-genome with 15 other species based on orthologous relationships.

Blue numbers at the nodes are divergence time to present (MYA). Red dots represent the calibration time of *B. distachyon*-*O. sativa* and *A. thaliana*-*P. trichocarpa* that were derived from the previously analysis.

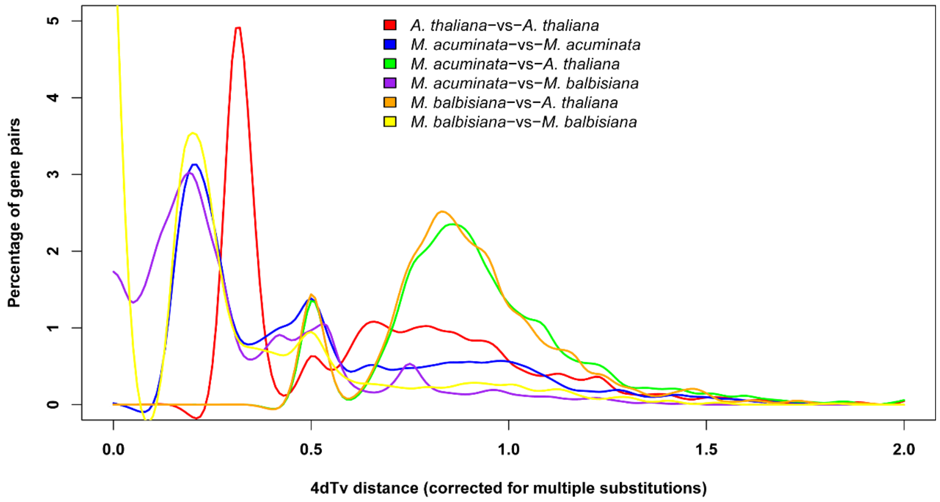

Supplementary Figure 6. Distribution of the 4dTv distance between duplicated genes in syntenic blocks among *M. acuminata*, *M. balbisiana* and *A. thaliana*.

The purple and yellow line represents the 4dTv distribution of A-genome and B-genome respectively.

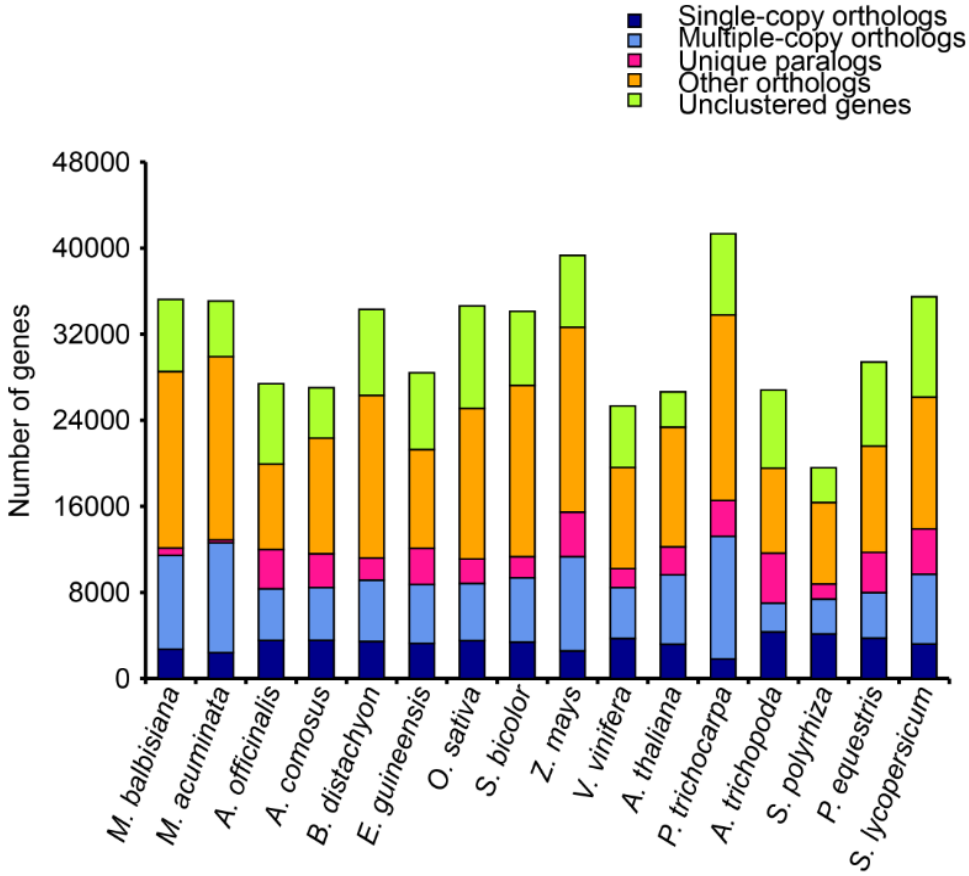

Supplementary Figure 7. Gene numbers in each category that were defined by OrthoMCL.

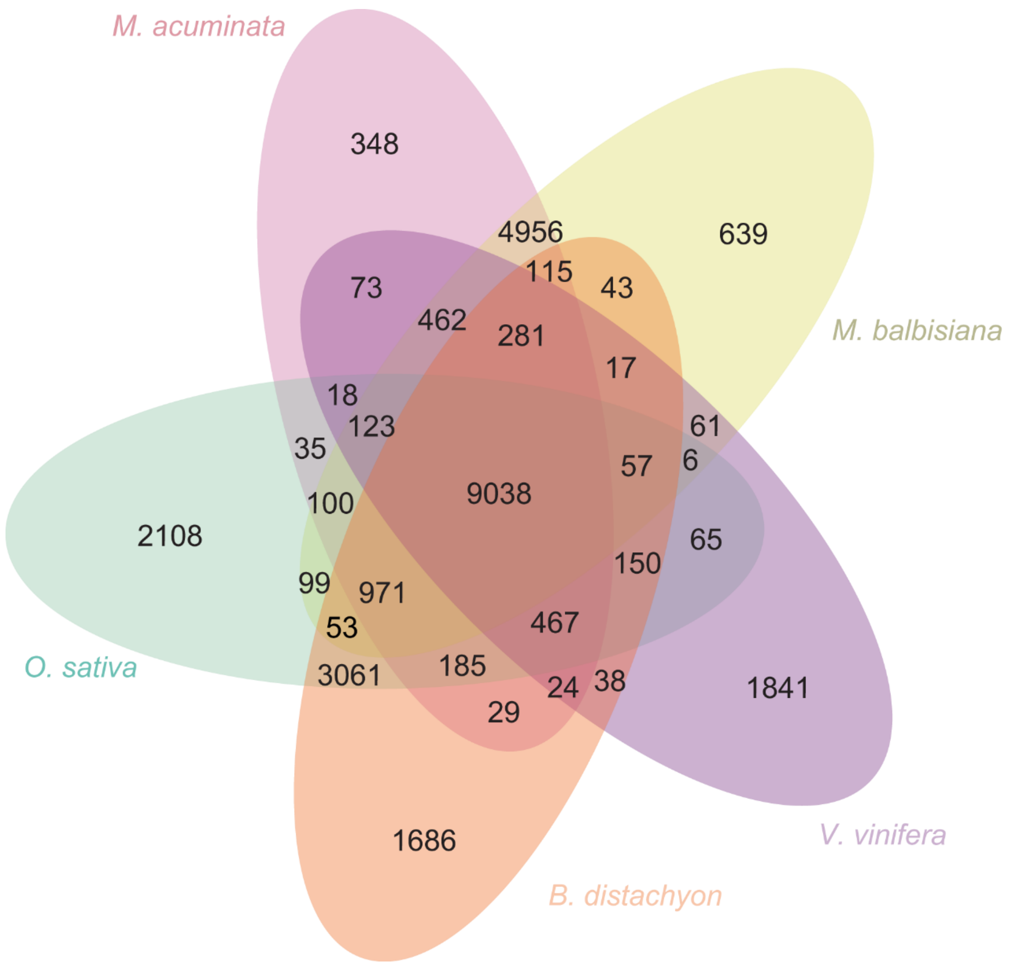

Supplementary Figure 8. Venn diagram showing the shared orthologous groups among *M. balbisiana*, *M. acuminata*, O. *sativa*, *B. distachyon*, and *V. vinifera*.

The number within the circles indicates the number of gene families in each cluster.

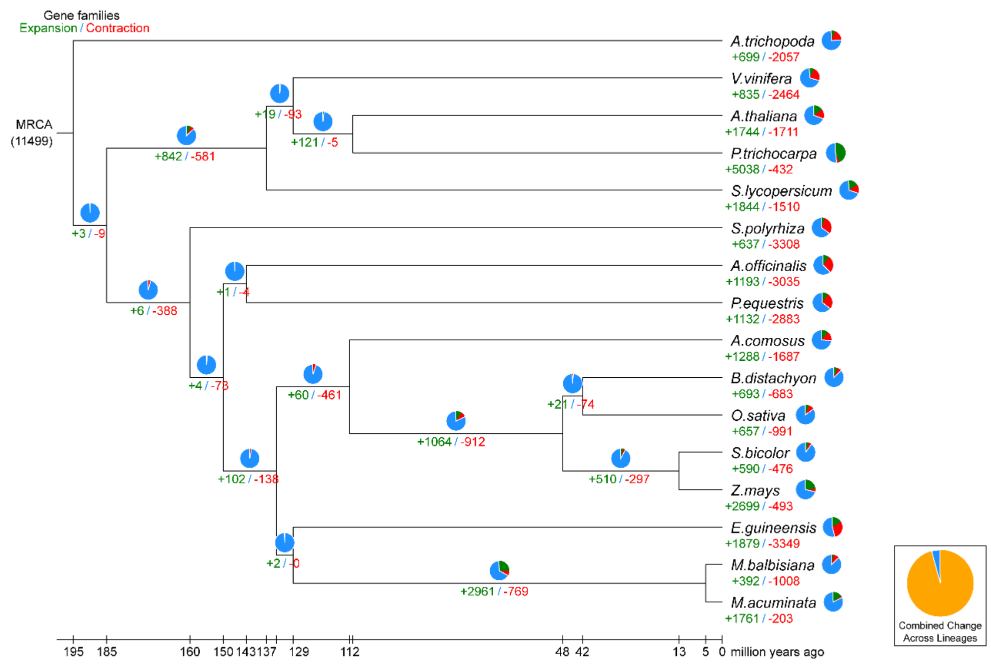

Supplementary Figure 9. Phylogenetic relationship and the expansion and contraction of gene families.

Gene family expansions are indicated in green, and gene family contractions are indicated in red. The corresponding proportions among the total changes are shown as pie charts using the same colors. Blue portions of the pie charts represent conserved gene families.

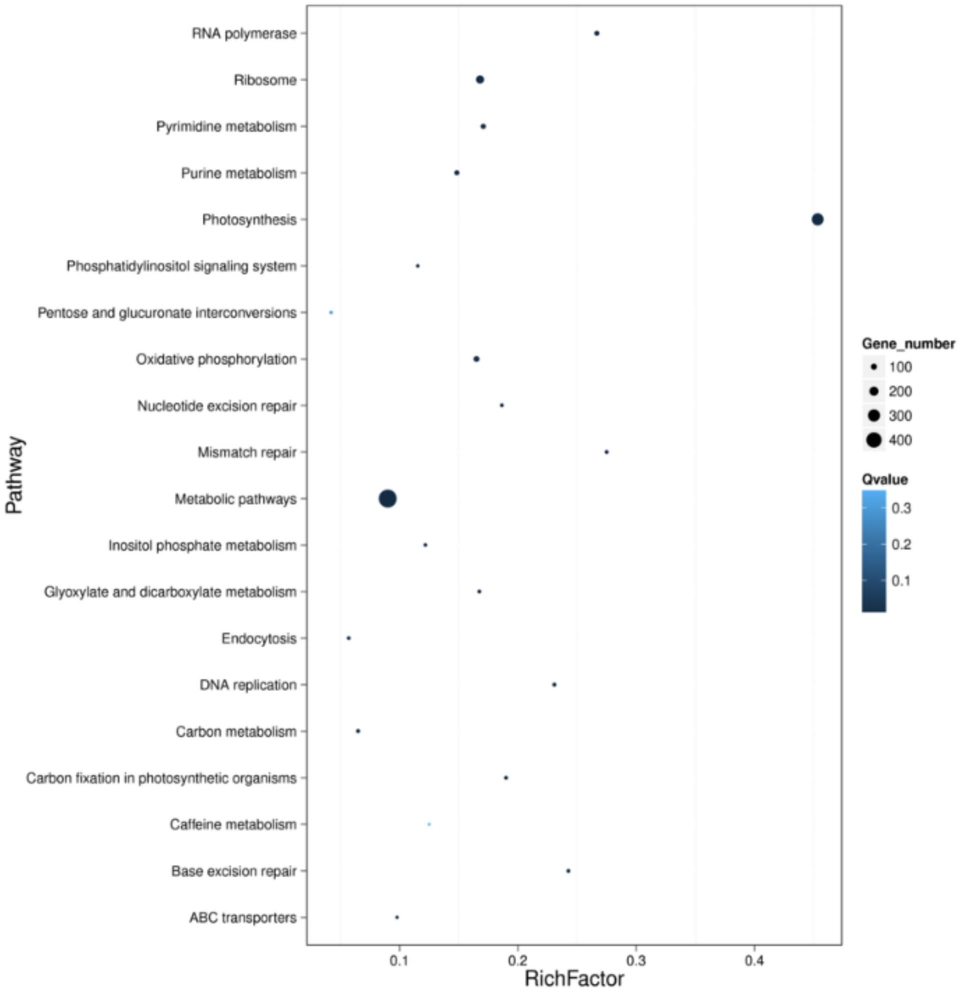

Supplementary Figure 10. The KEGG pathway enrichment analysis of the significantly expanded gene families in the B-genome. A total of 757 (sample size) genes were used in enrichment analysis. The gene set enrichment was analyzed using hypergeometric testing. Q-value was calculated using FDR (False Discovery Rate) adjustment method for correcting multiple hypothesis testing.

Top 20 pathways are shown. Q-values represent the significance of enrichment. Circles indicates the target genes, and the size is proportional to the number of genes.

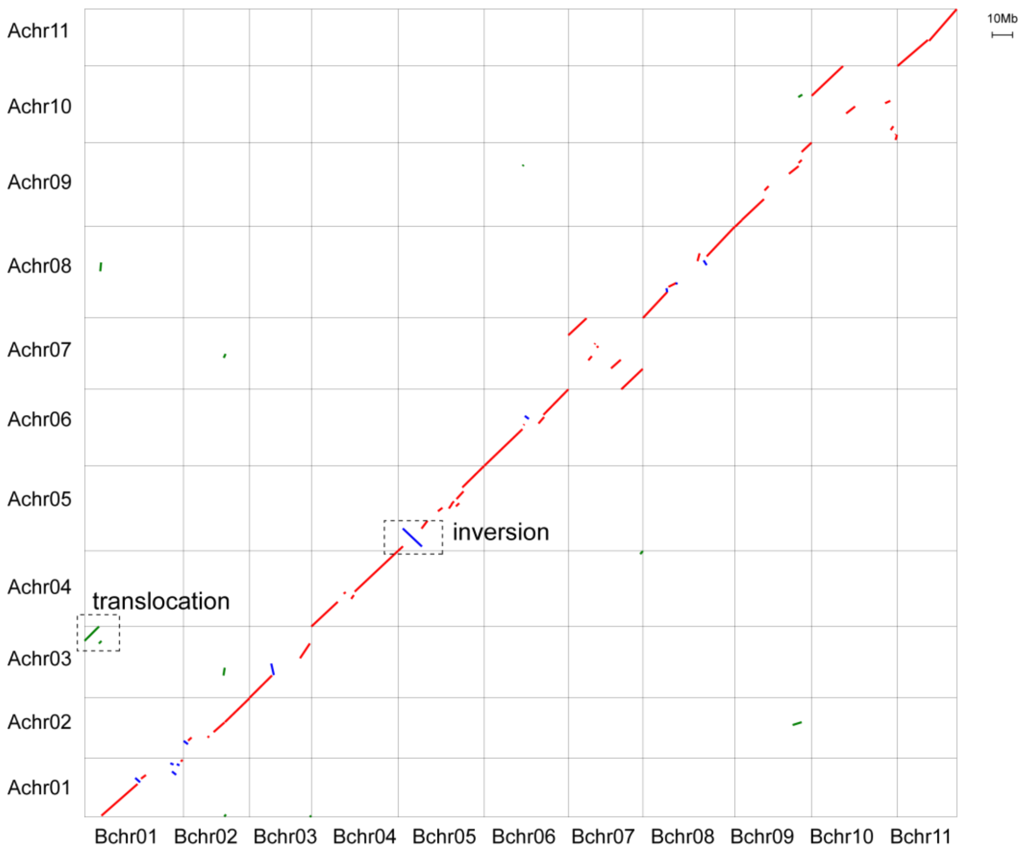

.

Supplementary Figure 11. Syntenic relationship between *M. balbisiana* and *M. acuminata* genomes.

The relationship of the chromosomes between the two species are shown. Red line represents alignment blocks, blue line represents inversion and green line represents translocation blocks.

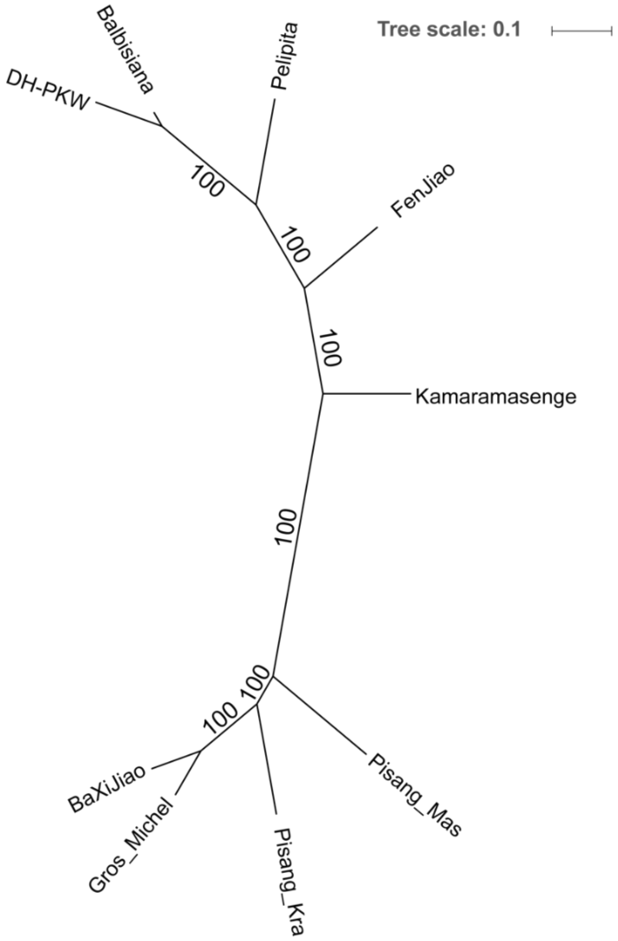

Supplementary Figure 12. Phylogenetic relationships of the nine resequenced banana accessions based on genotyping data.

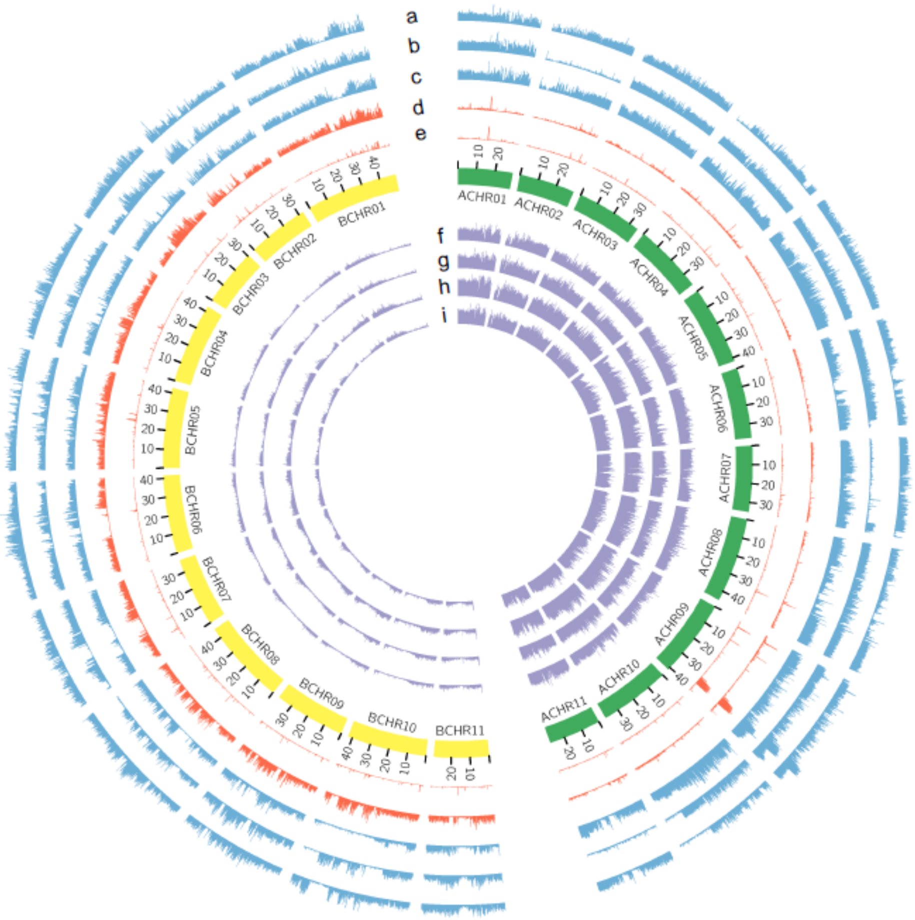

Supplementary Figure 13. The SNP density distributions with 50-kb non overlapping sliding windows of the nine resequencing samples on A- and B-genome (*M. acuminata* and *M. balbisiana*). The samples are represents as follows : a: Fen Jiao (genome group: ABB), b: Pelipita (genome group: ABB), c: Kamaramasenge (genome group: AAB), d: Balbisiana (genome group: BB), e: DH_PKW (genome group: BB), f: Pisang Kra (genome group: AA), g: Pisang Mas (genome group: AA), h: Gros_Michel (genome group: AAA), i: BaXiJiao (genome group: AAA).

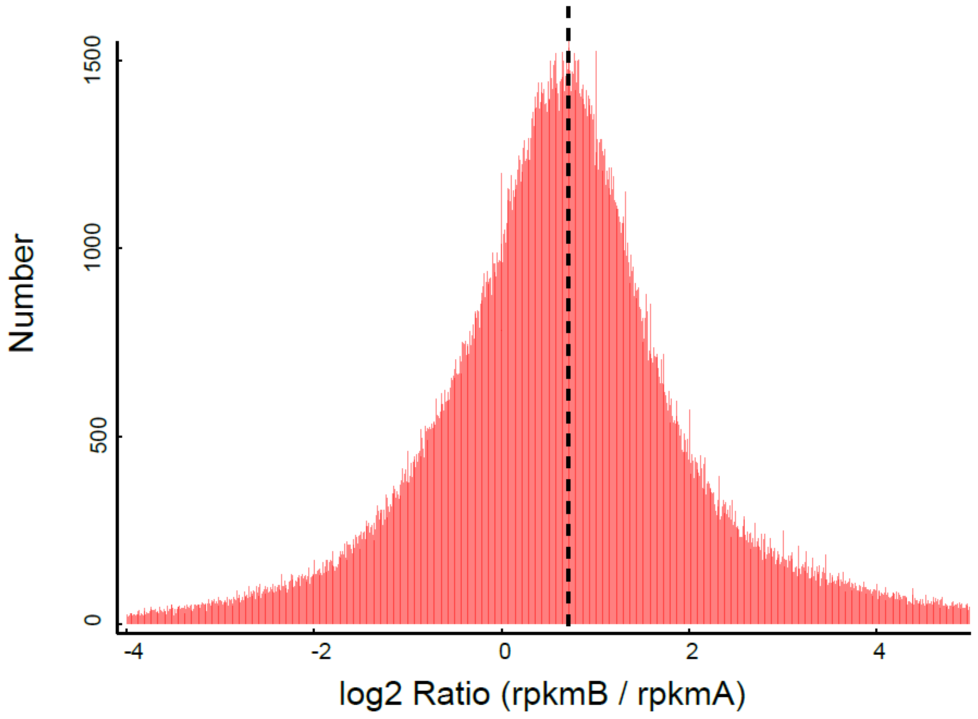

Supplementary Figure 14. The log2 (RPKM B / RPKM A) expression distribution of all homoeologous gene pairs between A- and B-subgenomes in FJ.

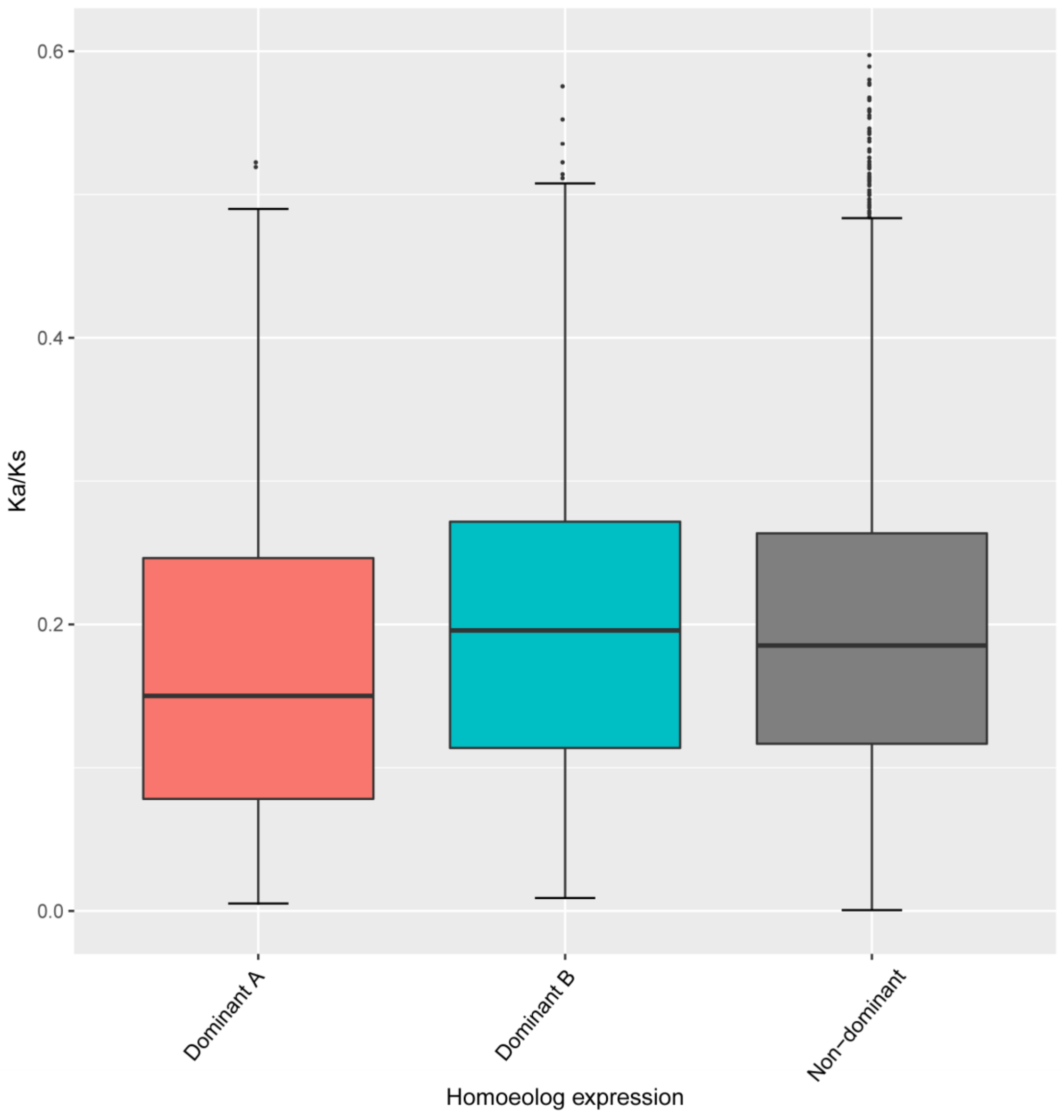

Supplementary Figure 15. Box plots of Ka/Ks values of homoeologs pairs with expression dominance in FJ. The minima, maxima, centre, upper and lower quartiles were shown in the figure.

There are 243, 1,777 and 7,804 homoeologs pairs with expression dominance in A-subgenome, B-subgenome, and non-expression dominance respectively, and they are defined as Dominant A, Dominant B, and Non-dominant, respectively.

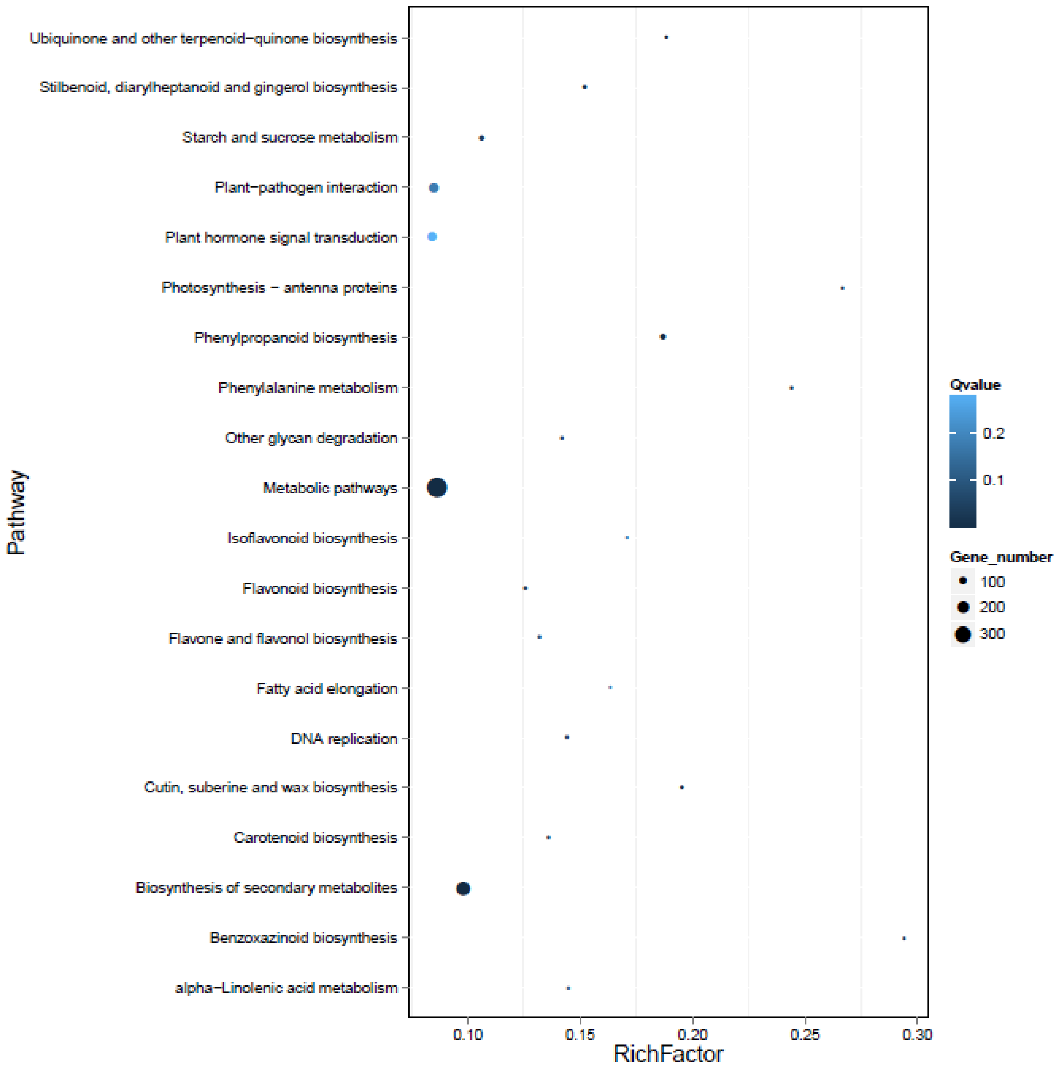

Supplementary Figure 16. KEGG pathway enrichment analysis for the genes in the co-expression network of the A-subgenome. A total of 1,418 (sample size) genes were used in enrichment analysis. The gene set enrichment was analyzed using hypergeometric testing. Q-value was calculated using FDR (False Discovery Rate) adjustment method for correcting multiple hypothesis testing.

The top 20 pathways were shown. Q-values represent the significance of enrichment. Circles indicate the target genes and the size is proportional to the number of genes.

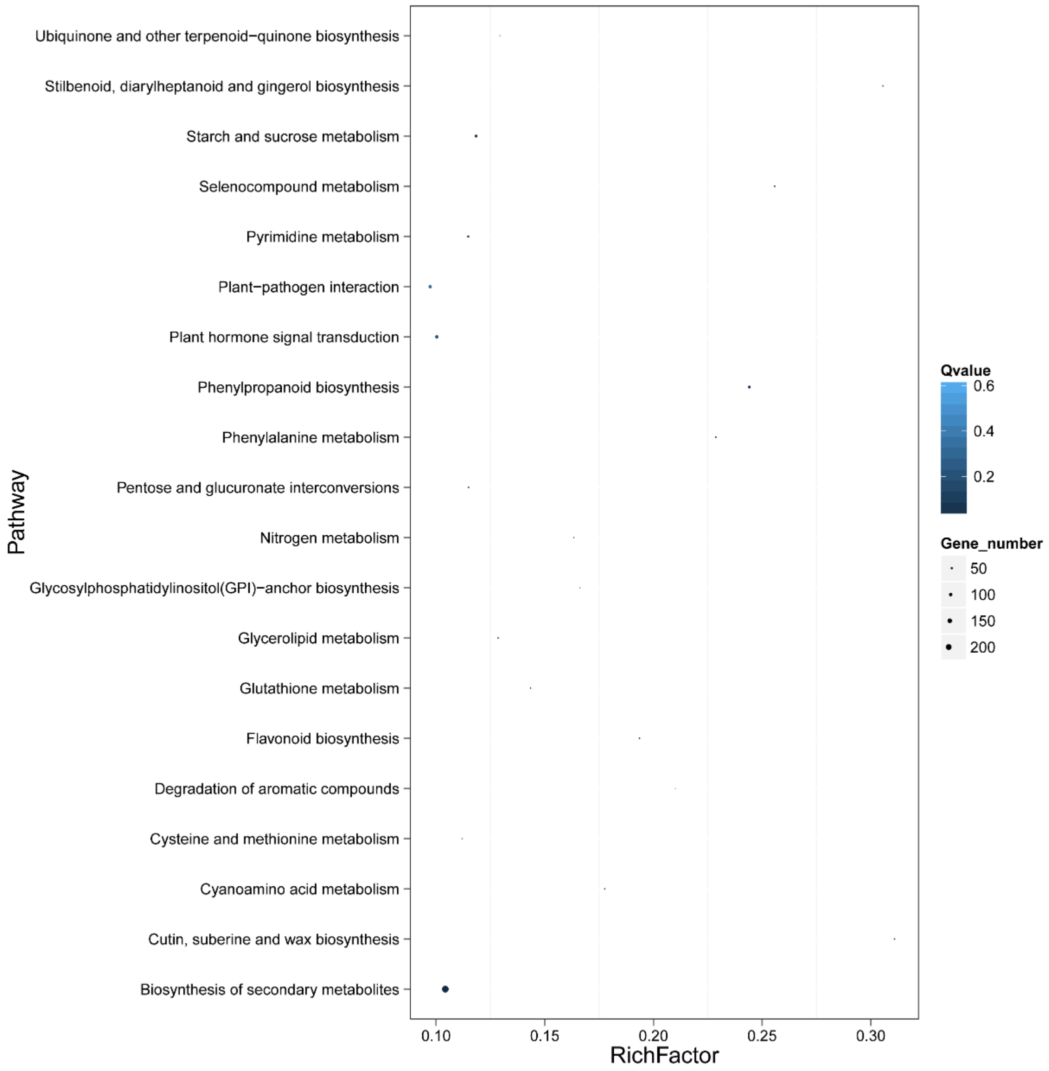

Supplementary Figure 17. KEGG pathway enrichment analysis for the genes in the co-expression network of the B subgenome. A total of 2,028 (sample size) genes were used in enrichment analysis. The gene set enrichment was analyzed using hypergeometric testing. Q-value was calculated using FDR (False Discovery Rate) adjustment method for correcting multiple hypothesis testing.

The top 20 pathways were shown. Q-values represent the significance of enrichment. Circles indicate the target genes and the size is proportional to the number of genes.

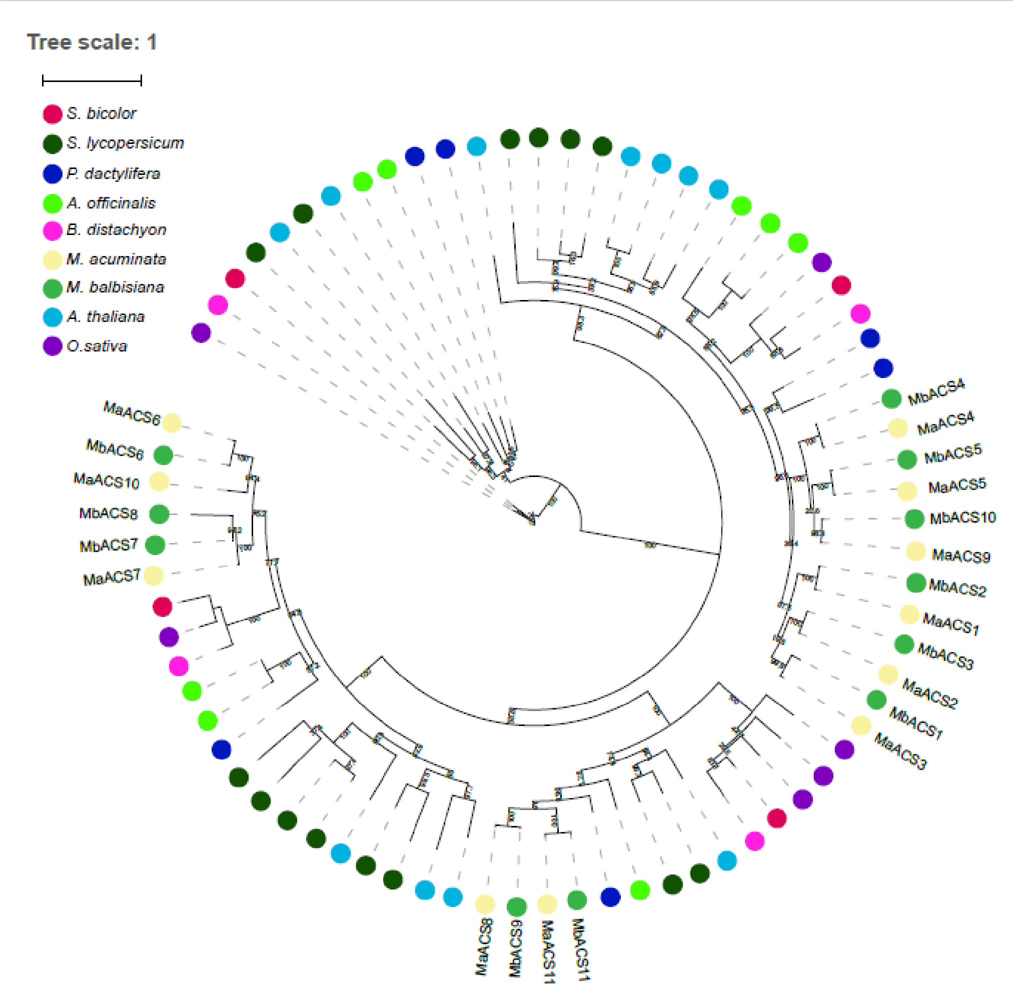

Supplementary Figure 18. Phylogenetic analysis of the ACS gene family among nine species.

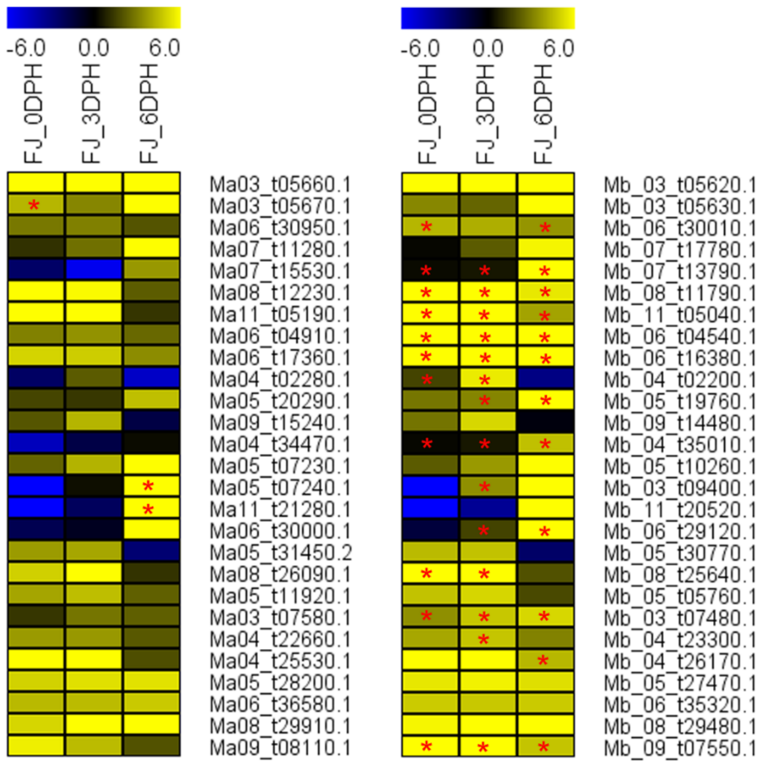

Supplementary Figure 19. The expression dominance (Log2 based RPKM) of homoeolog gene pairs that are related to fruit ripening between the A- and B-subegenomes of FJ.

Horizontal genes in the heat map indicate homologous gene pairs between the A- and B-subgenomes. Asterisks indicate the dominant homoeolog expression between the A- and B-subgenomes of FJ. Days post-harvest (DPH) are fruit ripening stages.

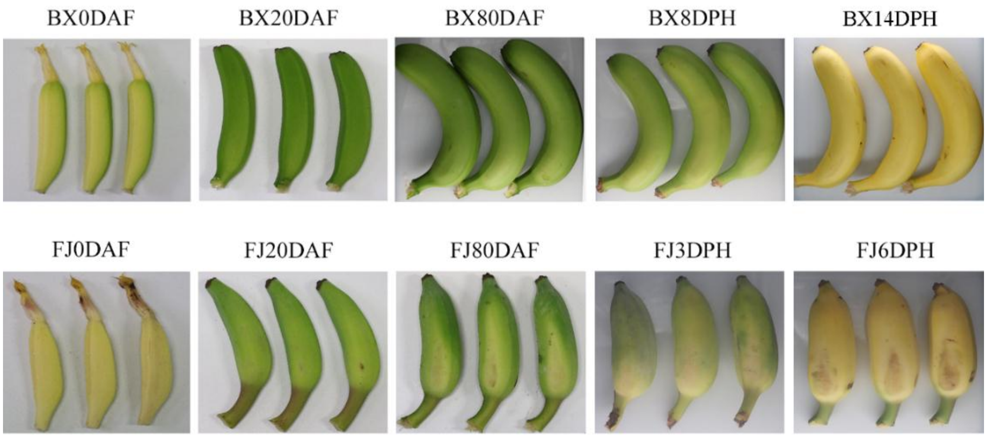

Supplementary Figure 20. Phenotype of BX and FJ at different stages of fruit development and ripening. DAF, days after flower; DPH, days postharvest. The experiment was repeated three times independently with similar results.

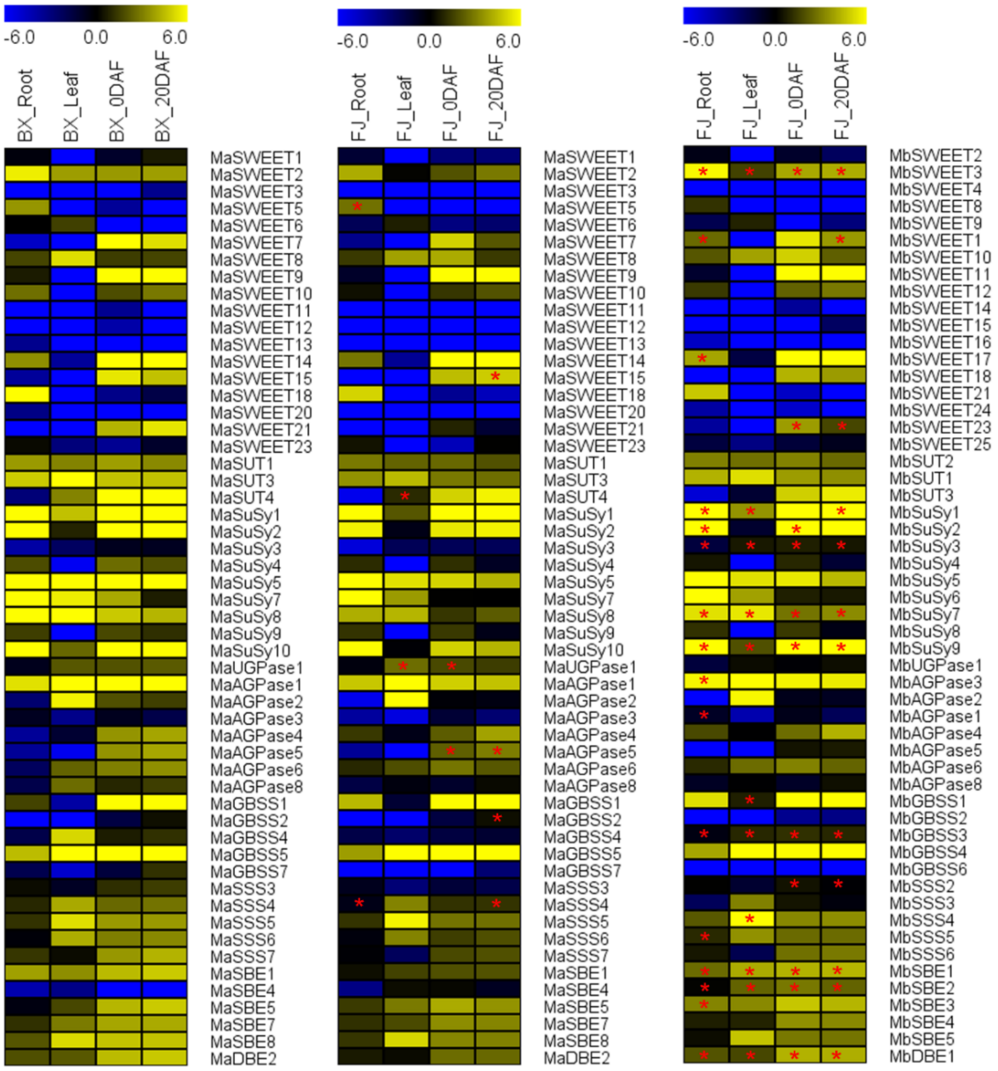

Supplementary Figure 21. The expression dominance (Log2 based RPKM) of homoeolog gene pairs that are related to starch synthesis pathway within the roots, leaves, and fruits between the A- and B-subegenomes of FJ.

Horizontal genes in the heat map indicate homoeolog gene pairs between the A- and the B-subgenomes. Asterisks indicate the dominant homoeolog expression between the A- and B-subgenomes of FJ. DAF, days after flowering.

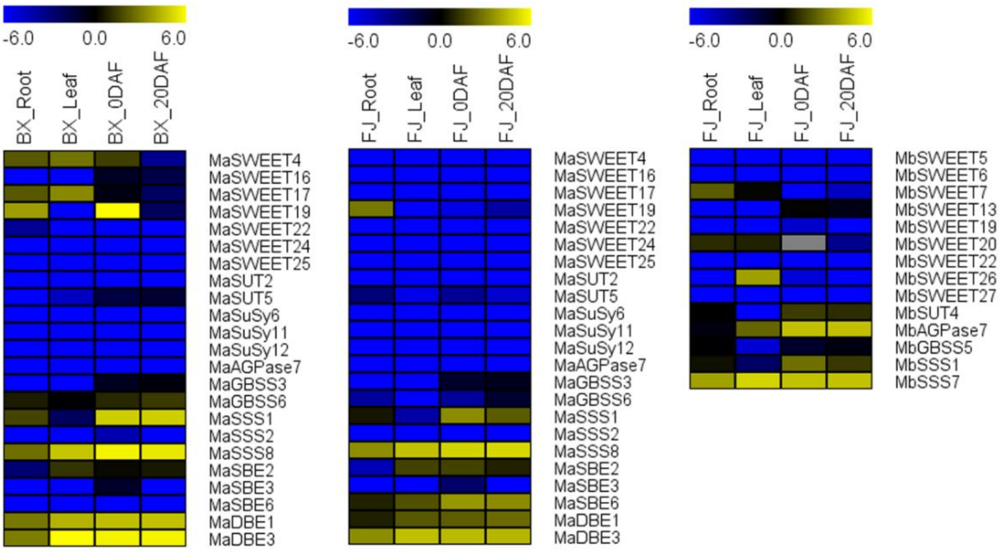

Supplementary Figure 22. Expression patterns (Log2 based RPKM) of genes in the starch synthesis pathway unique to the A- or B- genomes within the roots, leaves, and fruits.

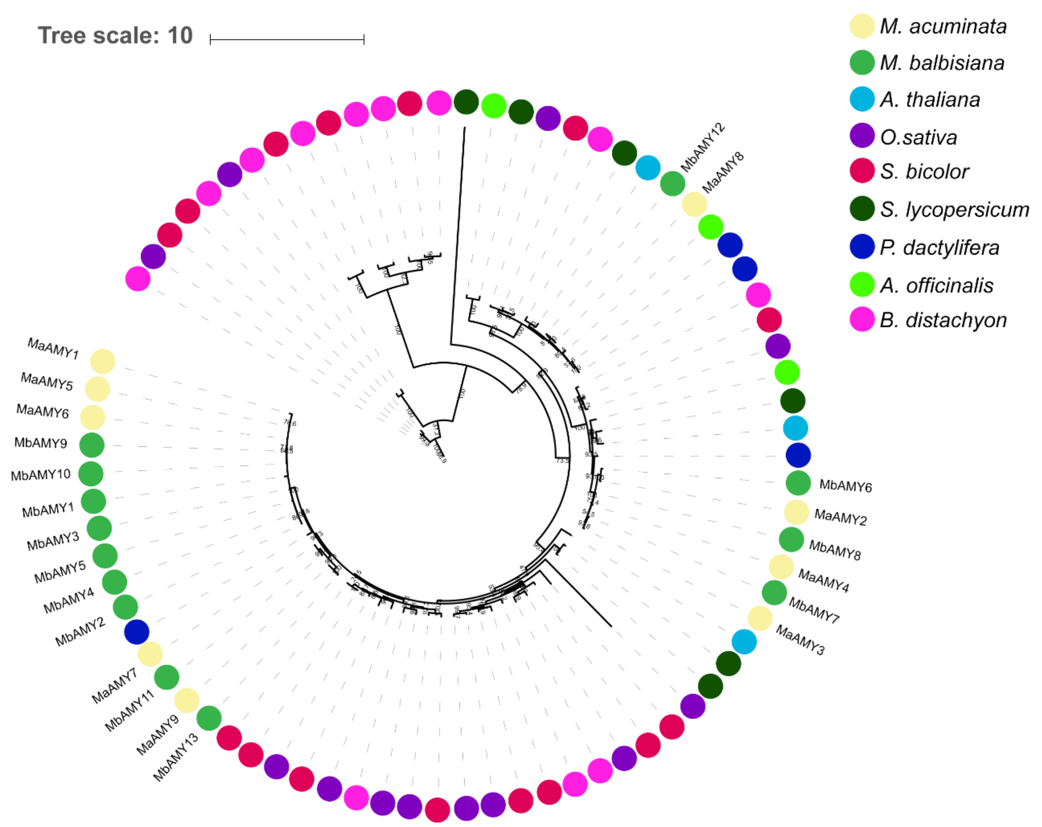

Supplementary Figure 23. Phylogenetic analysis of the AMY gene family among nine species.

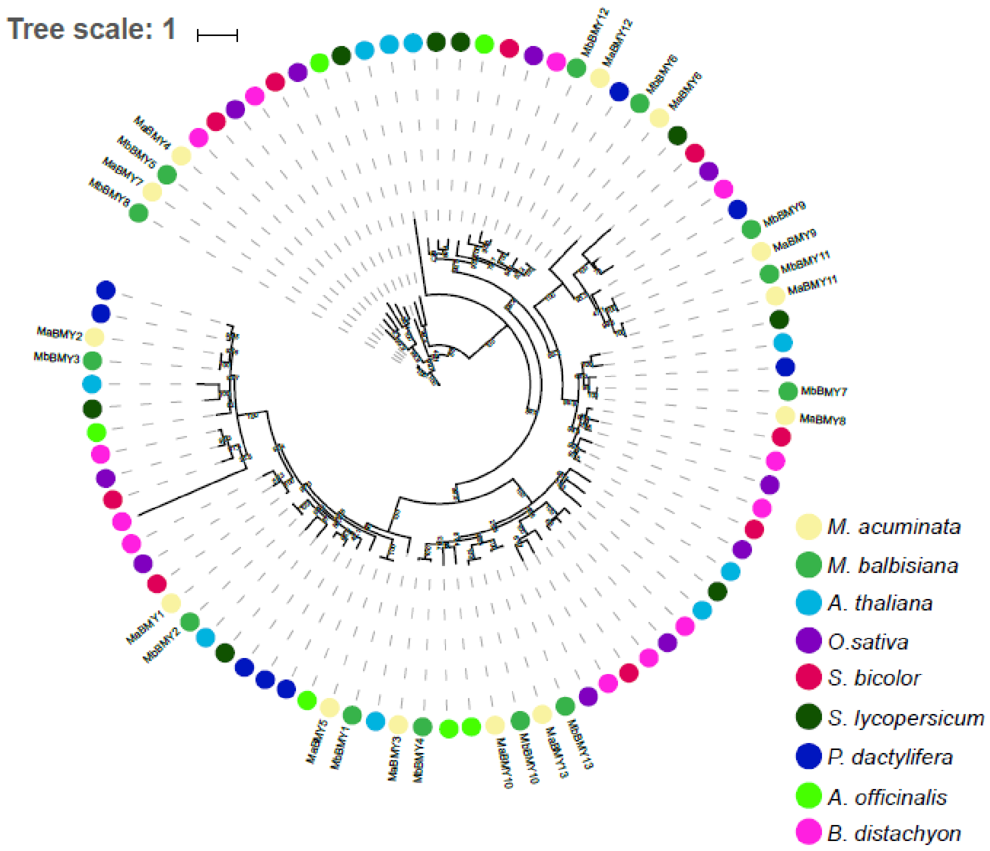

Supplementary Figure 24. Phylogenetic analysis of the BMY gene family among nine species.

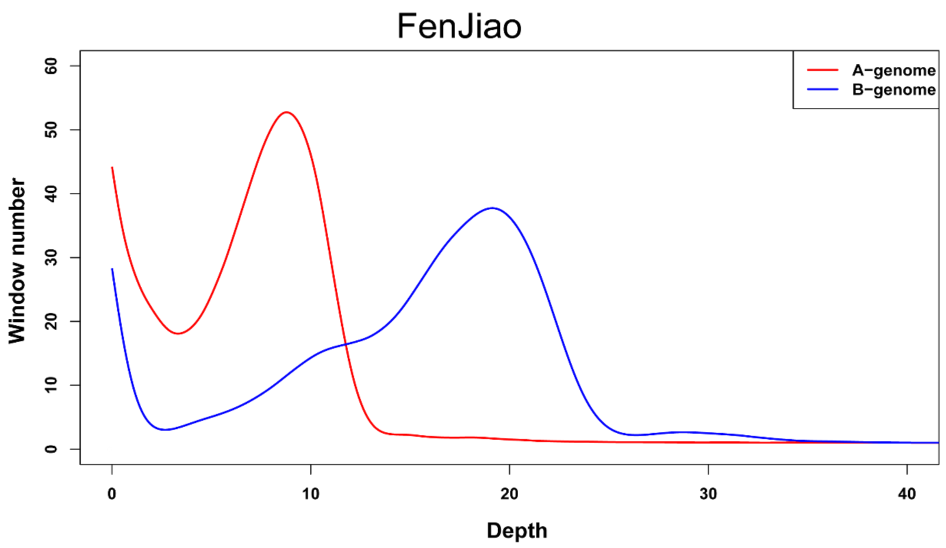

Supplementary Figure 25. Depth distributions for 10-kb non overlapping sliding windows in FenJiao (genome group: ABB). The red line represents the depth of A-subgenome and blue line represents the depth of B-subgenome.

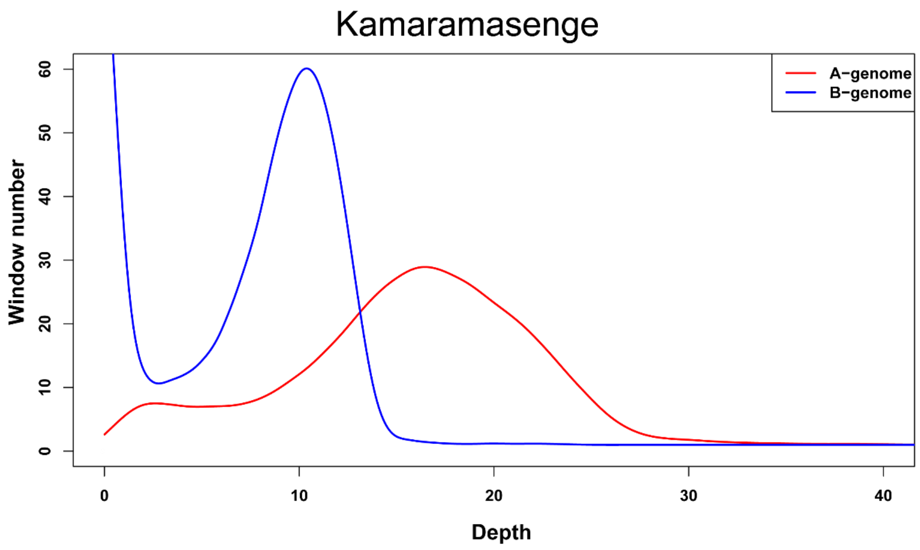

Supplementary Figure 26. Depth distributions for 10-kb non overlapping sliding windows in Kamaramasenge (genome group: AAB). The red line represents the A subgenome and the blue line represents the B subgenome.

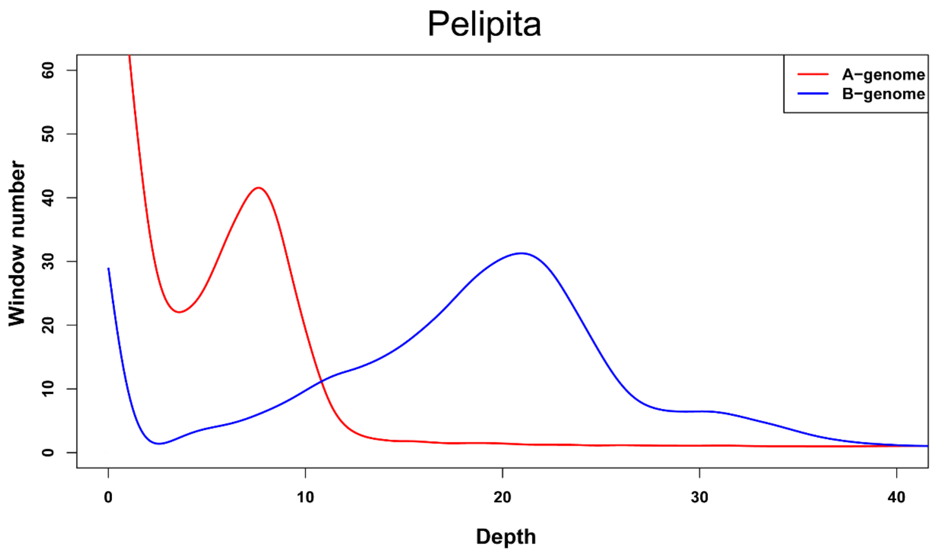

Supplementary Figure 27. Depth distributions for 10-kb non overlapping sliding windows in Pelipita (genome group: ABB). The red line represents the A subgenome and the blue line represents the B subgenome.

Supplementary Tables

Supplementary Table 1. Overview of sequencing data in *Musa balbisiana* (DH-PKW).

Supplementary Table 2. Overview of genome assembly of *Musa balbisiana*. (DH-PKW) and *Musa acuminata*.

Supplementary Table 3. Overview of assembly anchoring on the 11 pseudo-molecules of *Musa balbisiana* and *Musa acuminata*.

Supplementary Table 4. Statistics of repeat contents in the assembled B-genome (*M. balbisiana*) and A-genome (*M. acuminata*).

Supplementary Table 5. General statistics of predicted protein-coding genes.

Supplementary Table 6. Transcription factor families in 7 plant genomes.

Supplementary Table 7. Gene families in 16 plant genomes.

Supplementary Table 8. The statistics of gene family expansion in A-genome.

Supplementary Table 9. The statistics of gene family expansion in B-genome.

Supplementary Table 10. KEGG enrichment analysis of the significantly expanded gene families in B-genome using hypergeometric test.

Supplementary Table 11. Syntenic blocks between *M. balbisiana* and *M. acuminata* and among other species.

Supplementary Table 12. The synteny statistics of *M. balbisiana* and *M. acuminata* to 12 ancestral blocks.

Supplementary Table 13. Banana accessions used for resequencing analysis.

Supplementary Table 14. Statistics of banana resequencing data.

Supplementary Table 15. Homeologous exchanges in the three triploid samples.

Supplementary Table 16. Summary of SNPs identified in resequencing accessions.

Supplementary Table 17. Summary of Indels identified in resequencing accessions.

Supplementary Table 18. Summary of SVs identified in resequencing accessions.

Supplementary Table 19. KEGG pathway enrichment of the 83 gene families that are significantly expanded in the A-genome (and conversely contracted in the B-genome) using hypergeometric test.

Supplementary Table 20. KEGG pathway enrichment of the 33 gene families that are significantly expanded in the B-genome (and conversely contracted in the A-genome) using hypergeometric test.

Supplementary Table 21. Statistics of samples used for RNA-Seq sequencing.

Supplementary Table 22. Homoeolog gene pairs between *M. acuminata* and *M.*

balbisiana.

Supplementary Table 23. Expression dominance of homoeolog gene pairs in A subgenome/ B subgenome of triploid FJ.

Supplementary Table 24. KEGG enrichment analysis of the genes with expression dominance in the B-subgenome using hypergeometric test.

Supplementary Table 25. KEGG pathway enrichment of the genes which interacted with expression dominance genes of A-subgenome using hypergeometric test.

Supplementary Table 26. KEGG pathway enrichment of genes which interacted with expression dominance genes of B-subgenome using hypergeometric test.

Supplementary Table 27. The name and accession number of the genes related to ethylene biosynthesis in A- and B-genome.

Supplementary Table 28. The number of genes related to ethylene biosynthesis in various species.

Supplementary Table 29. Homoeolog gene pairs related to ethylene biosynthesis between A and B-genome.

Supplementary Table 30. The expression data (Log2 based RPKM) of the genes related to ethylene biosynthesis in BX variety.

Supplementary Table 31.The expression data (Log2 based RPKM) of the genes related to ethylene biosynthesis in A subgenome of FJ variety.

Supplementary Table 32.The expression data (Log2 based RPKM) of the genes related to ethylene biosynthesis in B subgenome of FJ variety.

Supplementary Table 33. Expression data of 28 homoeolog gene pairs related to fruit

ripening between A- and B-subgenome in 7 samples (each sample has two replicates) of FJ variety.

Supplementary Table 34.Overview of homoeolog gene pairs expressin dominance related to fruit ripening between A- and B-subgenome in FJ variety.

Supplementary Table 35. Synteny analysis of ACO genes between *M. acuminata* and *M. balbisiana*.

Supplementary Table 36.The expression data (Log2 based RPKM) of homoeolog gene pairs related to fruit ripening between A- and B-subgenome in FJ variety.

Supplementary Table 37. Overview of homoeolog gene pairs related to fruit ripening between A- and B-subgenome in FJ variety.

Supplementary Table 38.The expression data (Log2 based RPKM) of the ACO genes expanded in B-subgenome of FJ variety.

Supplementary Table 39. The name and accession number of the genes related to starch metabolism in A- and B-genomes.

Supplementary Table 40. The number of genes related to starch metabolism in various species.

Supplementary Table 41. The expression data (Log2 based RPKM) of the genes related to starch biosynthesis in BX variety.

Supplementary Table 42. The expression data (Log2 based RPKM) of the genes related to starch biosynthesis in A-subgenome of FJ variety.

Supplementary Table 43. The expression data (Log2 based RPKM) of the genes related to starch biosynthesis in B-subgenome of FJ variety.

Supplementary Table 44. Expression data of 54 homoeolog gene pairs related to starch biosynthesis between A- and B-subgenomes in 4 samples (each sample has two replicates) of FJ variety.

Supplementary Table 45. Overview of homoeolog gene pairs express in dominance related to starch biosynthesis between A- and B-subgenomes in FJ variety.

Supplementary Table 46. The expression data (Log2 based RPKM) of the genes related to starch degradation in BX variety.

Supplementary Table 47. The expression data (Log2 based RPKM) of the genes related to starch degradation in A-subgenome of FJ variety.

Supplementary Table 48. The expression data (Log2 based RPKM) of the genes related to starch degradation in B-subgenome of FJ variety.

Supplementary Table 49. Expression data of 21 homoeolog gene pairs related to starch degradation between A- and B-subgenomes in 3 samples (each sample has two replicates) of FJ variety.

Supplementary Table 50. Overview of homoeolog gene pairs expression dominance related to starch degradation between A-and B-subgenomes.

Supplementary Table 51. Genes of ethylene biosynthesis and starch metabolism in various species used for homolog-based prediction.

Supplementary Table 52. Non-coding RNA annotation of *M. balbisiana*.
