# Supplementary material for: Musa balbisiana genome reveals subgenome evolution and functional divergence
Source: Nat Plants. 2019 Jul 15;5(8):810–21. doi: 10.1038/s41477-019-0452-6 (PMC6784884; doi:10.1038/s41477-019-0452-6)
Supplement: Supplementary file 2 — Reporting Summary [file 41477_2019_452_MOESM2_ESM.pdf]

nature research

Corresponding author(s): Wei Hu, Zhiqiang Jin and Angélique D'Hont

Last updated by author(s): Apr 29, 2019

Reporting Summary

Nature Research wishes to improve the reproducibility of the work that we publish. This form provides structure for consistency and transparency in reporting. For further information on Nature Research policies, see Authors & Referees and the Editorial Policy Checklist.

Statistics

For all statistical analyses, confirm that the following items are present in the figure legend, table legend, main text, or Methods section.

n/a	Confirmed	
☐	☒	The exact sample size (*n*) for each experimental group/condition, given as a discrete number and unit of measurement
☐	☒	A statement on whether measurements were taken from distinct samples or whether the same sample was measured repeatedly
☐	☒	The statistical test(s) used AND whether they are one- or two-sided<br>*Only common tests should be described solely by name; describe more complex techniques in the Methods section.*
☒	☐	A description of all covariates tested
☐	☒	A description of any assumptions or corrections, such as tests of normality and adjustment for multiple comparisons
☐	☒	A full description of the statistical parameters including central tendency (e.g. means) or other basic estimates (e.g. regression coefficient) AND variation (e.g. standard deviation) or associated estimates of uncertainty (e.g. confidence intervals)
☐	☒	For null hypothesis testing, the test statistic (e.g. *F*, *t*, *r*) with confidence intervals, effect sizes, degrees of freedom and *P* value noted<br>*Give P values as exact values whenever suitable.*
☒	☐	For Bayesian analysis, information on the choice of priors and Markov chain Monte Carlo settings
☒	☐	For hierarchical and complex designs, identification of the appropriate level for tests and full reporting of outcomes
☒	☐	Estimates of effect sizes (e.g. Cohen's *d*, Pearson's *r*), indicating how they were calculated

Our web collection on statistics for biologists contains articles on many of the points above.

Software and code

Policy information about availability of computer code

Data collection	Homolog data (homolog species genome and gene set) was downloaded from public databases: NCBI, JGI, Uniprot, KEGG, GO and NR. Genome data of Musa acuminata was downloaded from http://banana-genome-hub.southgreen.fr/. Genomes of Musa balbisiana (DH-PKW) and nine different accessions, and transcriptome data of Baxijiao and Fenjiao were sequenced by ourselves in this research.
Data analysis	We used lots of software for data analysis in this paper, and all data and software used was described in Methods section of manuscript.<br>Genome assembly: wtdbg v1.2.8, SSPACE v3, Arrow, LACHESIS, HiC-Pro v2.8.1<br>Evaluation of assembly quality: BUSCO v3, BLAT v35, BWA v0.712<br>Repeat annotation: RepeatMasker v4.0.6, RepeatProteinMask, Repbase v21.01, Piler v1.0, RepeatScout v1.0.5, LTR-FINDER v1.0.5, Tandem Repeats Finder v4.09<br>Gene structure annotation: Blast v2.2.26, Augustus v3.2.1, SNAP, HISAT2 v2.0.1-beta, StringTie v1.2.1, PASA_lite, MAKER v3.31.8<br>Genome annotation completeness: BUSCO v3<br>Gene function annotation: BLAST v2.2.26, InterProScan v5.16<br>ncRNA annotation: tRNAscan-SE v1.23, INFERNAL, BLAST v2.2.26<br>Gene family analysis: OrthoMCL v1.4, CAFE v2.1, blast v2.2.26<br>Phylogentic analysis: PAML package v4.4, MrBayes v3.1.2<br>Transcription factor prediction: iTAK v1.5<br>Resequencing analysis: bwa 0.7.12, GATK v3.3-0, Breakdancer<br>Nucleotide diversity: VCFtools v0.1.13<br>Genome syntenic analysis: blast v2.2.26, MCscan v1.5.1, KaKs_Calculator v2.0<br>Gene co-expression network analysis:R platform v3.2.2, WGCNA package v1.47<br>RNA-Seq analysis: SOAPaligner/SOAP2 v2.21, DEGseq

For manuscripts utilizing custom algorithms or software that are central to the research but not yet described in published literature, software must be made available to editors/reviewers. We strongly encourage code deposition in a community repository (e.g. GitHub). See the Nature Research guidelines for submitting code & software for further information.

Data

Policy information about availability of data

All manuscripts must include a data availability statement. This statement should provide the following information, where applicable:

- Accession codes, unique identifiers, or web links for publicly available datasets
- A list of figures that have associated raw data
- A description of any restrictions on data availability

Raw sequence reads for gene assembly and gene expression for all samples were deposited in the Sequence Read Archive (SRA) of the National Center for Biotechnology Information (NCBI) under the BioProject (PRJNA432894). Genome assembly and annotation of DH-PKW was submitted to NCBI (PYDT00000000). A full data availability statement is included in the manuscript.

Field-specific reporting

Please select the one below that is the best fit for your research. If you are not sure, read the appropriate sections before making your selection.

☒ Life sciences ☐ Behavioural & social sciences ☐ Ecological, evolutionary & environmental sciences

For a reference copy of the document with all sections, see nature.com/documents/nr-reporting-summary-flat.pdf

Life sciences study design

All studies must disclose on these points even when the disclosure is negative.

Sample size	One double haploid (DH-PKW) sample from M. balbisiana was used for genome assembly. Nine different genotypes (AAA, ABB, AA, BB, AAB) of banana accessions were used for resequencing. Two cultivated varieties of BaXiJiao and FenJiao were used for transcriptomic analysis and forty samples were collected from different tissues and treatments including development and postharvest ripening process of fruits, and osmotic, salt and low temperature treatments. Two biological replicates were used for each sample.
Data exclusions	N/A
Replication	For gene expression profiling of BaXijiao and FenJiao, we produced RNA-seq data of fruits, roots, and leaves with two biological replicates.
Randomization	N/A
Blinding	N/A

Reporting for specific materials, systems and methods

We require information from authors about some types of materials, experimental systems and methods used in many studies. Here, indicate whether each material, system or method listed is relevant to your study. If you are not sure if a list item applies to your research, read the appropriate section before selecting a response.

Materials & experimental systems

n/a	Involved in the study	
☒	☐	Antibodies
☒	☐	Eukaryotic cell lines
☒	☐	Palaeontology
☒	☐	Animals and other organisms
☒	☐	Human research participants
☒	☐	Clinical data

Methods

n/a	Involved in the study	
☒	☐	ChIP-seq
☒	☐	Flow cytometry
☒	☐	MRI-based neuroimaging
